# Functional Assessments Used by Occupational Therapists with Older Adults at Risk of Activity and Participation Limitations: A Systematic Review

**DOI:** 10.1371/journal.pone.0147980

**Published:** 2016-02-09

**Authors:** Kylie Wales, Lindy Clemson, Natasha Lannin, Ian Cameron

**Affiliations:** 1 Ageing Work and Health Research Unit and Centre of Excellence in Population Ageing Research, Faculty of Health Sciences, University of Sydney, Lidcombe, NSW, Australia; 2 School of Allied Health, La Trobe University and Occupational Therapy Department, Alfred Health, Melbourne, VIC, Australia; 3 John Walsh Centre for Rehabilitation Research, Sydney Medical School Northern, University of Sydney, St Leonards, NSW, Australia; Cardiff University, UNITED KINGDOM

## Abstract

**Introduction:**

The use of functional assessments to evaluate patient change is complicated by a lack of consensus as to which assessment is most suitable for use with older adults. Objective: To identify and appraise the properties of assessments used to evaluate functional abilities in older adults.

**Methods:**

A systematic review of randomised controlled trials of occupational therapy interventions was conducted up to 2012 to identify assessments used to measure function. Two authors screened and extracted data independently. A second search then identified papers investigating measurement properties of each assessment. Studies from the second search were included if: i) published in English, ii) the assessment was not modified from its original published form, iii) study aim was to evaluate the quality of the tool, iv) and was original research. Translated versions of assessments were excluded. Measurement quality was rated using the COSMIN checklist and Terwee criteria.

**Results:**

Twenty-eight assessments were identified from the systematic search of occupational therapy interventions provided to older adults. Assessments were of varied measurement quality and many had been adapted (although still evaluated as though the original tool had been administered) potentially altering the conclusions drawn about measurement quality. Synthesis of best evidence established 15 functional assessments have not been tested in an older adult population.

**Conclusions:**

The Functional Autonomy Measurement System (SMAF) appears to be a promising assessment for use with older adults. Only two tools (the SMAF and the Assessment of Motor and Process Skills (AMPS)) were deemed to be responsive to change when applied to older adults. Health professionals should use functional assessments that have been validated with their population and in their setting. There are reliable and valid assessments to capture the functional performance of older adults in community and hospital settings, although further refinement of these assessments may be necessary.

## Introduction

Older adults commonly report restrictions in their ability to carry out meaningful everyday activities and often require assistance from others [1–3]. Standardised functional assessments provide a means for health professionals to analyse a person’s abilities to engage in and carry out daily activities [4]. The process of standardisation requires an assessment to have undergone rigorous evaluation and have established validity and reliability [4]. Such assessments can be used to determine the effectiveness of treatment, make comparisons across patient groups and amendments to treatment as needed [4, 5]. However, the routine use of standardised functional assessments in disciplines such as allied health is low and use of ‘in-house’ non-standardised assessments more common [6–11]. Reasons for limited use of standardised assessments include a lack of time and resources (including financial), and limited knowledge of the most suitable tool to use [6, 7, 11].

A prior systematic review of functional assessments appropriate for use in acute care, with older adults, concluded that there was significant variability in the assessment of function and that use of adapted (and untested) assessments occurred regularly [12]. In light of this variability, the authors recommended that future research should provide a clear definition of function and investigate which psychometrically robust assessment/s should be used with older adults [12].

While many professions assess function in health care, one profession with a central role is occupational therapy [13], whose major focus is to evaluate a person’s performance of everyday tasks (or ‘occupations’). Previous reviews of functional assessments in occupational therapy have supported the need for further investigation of their measurement properties [9, 14]. Functional assessments may be descriptive, evaluative (measuring change), discriminative and/or predictive of future functioning [4]. The purpose of assessments should be considered when reviewing measurement quality as certain qualities may be of higher value [4]. For example, responsiveness (the ability to detect change overtime) is an essential quality in evaluative tools.

This study addresses current gaps by first identifying standardised assessments used by occupational therapists with older adults to measure function and then appraising the measurement properties of each. Older adults were defined as being 70 years or older to reflect the increase in life expectancy in western countries [15]. Since therapists have a preference for in-house assessments, which are often ad-hoc and poorly documented [4], we developed a list of assessments available for use by reviewing randomised controlled trials (RCTs). We expected that assessments used in RCTs would more likely, but not necessarily, be more robust. Standardised functional assessments were defined as those which evaluated performance of the person within the activity and participation domains of the International Classification of Functioning, Disability and Health (ICF) [16]. Activity as defined by the World Health Organisation refers to the ‘execution of a task or activity by an individual’ and Participation ‘the involvement in a life situation’ [16]. These definitions reflect the areas in which occupational therapists provide intervention, that is, through engagement in meaningful everyday activities [17, 18]. Occupational therapy literature has further extended the definition of participation identifying the need to review the subjective experience and autonomy of participation [17]. Assessment tools which assessed bodily impairment, defined as problems in body function/structure were deemed to not reflect the intended focus of functional assessment and fell outside the scope of this study. For example, number of falls and related injuries would be considered a measurement of bodily impairment in this current study.

## Objectives

This study addressed the following research questions: i) (Phase 1) what standardised functional assessments have been used by occupational therapists to measure functional abilities in older adults in published randomised controlled trials?, ii) (Phase 2) what are the measurement properties of these identified assessments?, and iii) of these assessments which standardised functional assessment would be recommended for use with an older adult?

## Methods

### Search strategy and selection criteria

#### Phase 1: Identification of functional assessments used with older adults

**Fig 1:**
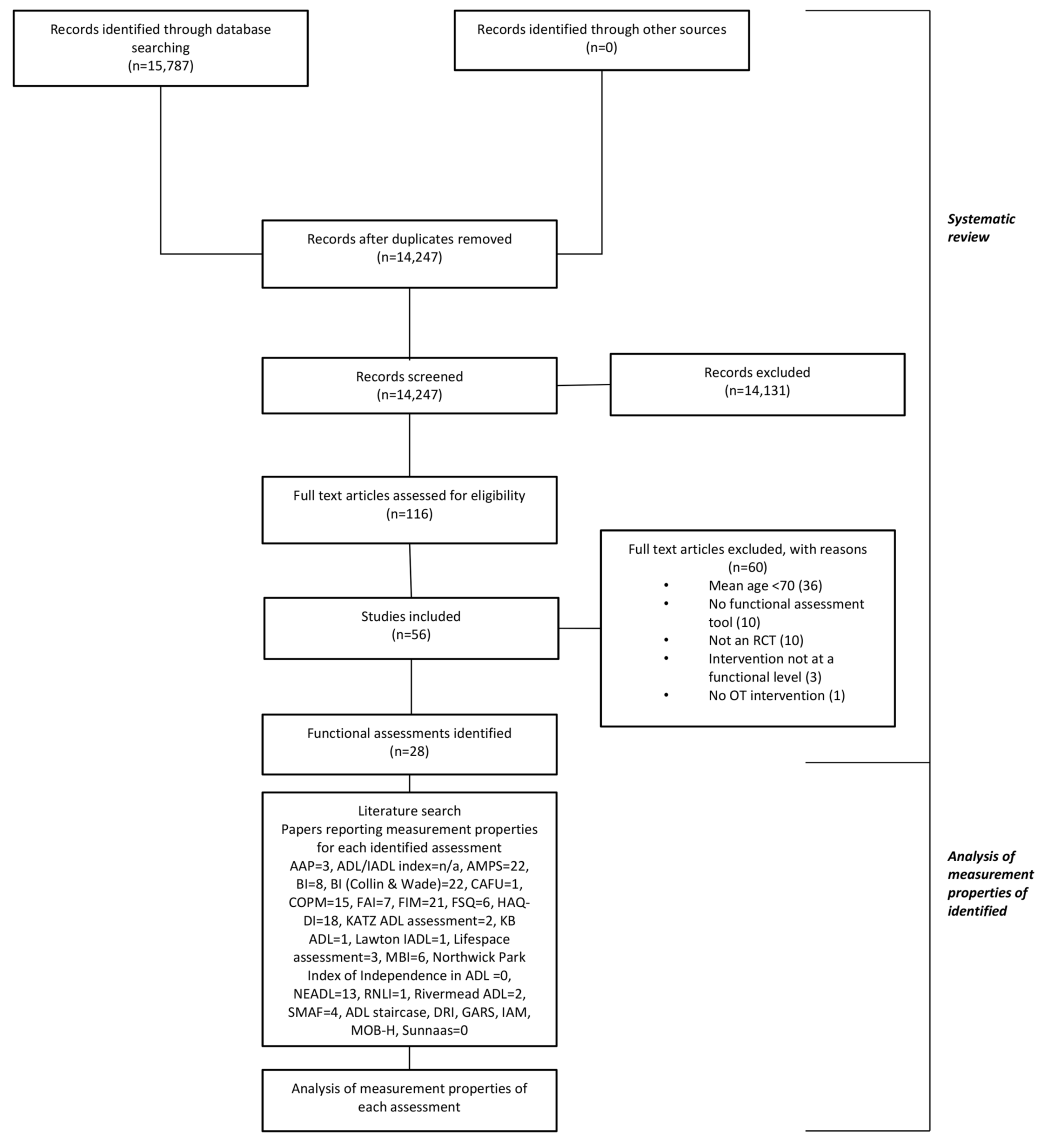
Systematic Review Process.

A database search of Medline, EBSCO (Cinhal) and OT Seeker was conducted up to February 2012 to identify the standardised functional assessments used to assess older adult participants in RCTs [19]. A pre-established search strategy was used [19] and databases were tested for search results prior to selection. Searches ended in February 2012 to allow for phase two searches and analysis to take place. Following removal of duplicates, title and or abstract of the papers were reviewed by one author [KW] who was experienced in the identification of occupational therapy (OT) based RCTs. Articles were included if: participants were 70 years and older, 2) an RCT design was used, 3) OT intervention was provided, and, 4) functional assessments were used. Full text papers were then screened independently by two authors [either KW, LC, NL or ID], first on abstract and then, if required, in full manuscript form. Differences of opinion were resolved through use of a third reviewer [either KW, LC, NL or ID]. For 21 papers authors were contacted to provide additional information not outlined in the full manuscript. A total of 56 papers were included. See Fig 1 for full search results. Further information on the methodology can be found in our protocol paper, which was published in lieu of a pre-registered protocol [19].

#### Phase 2: Measurement properties of identified functional assessments

In the second study phase, identified functional assessments were analysed for published information on their measurement properties. Search terms included the name of the assessment and all common abbreviations along with a search strategy designed to identify measurement properties in Medline, Embase and EBSCO (S1 Information). A number of databases were tested prior to selection and were retained in the search strategy if they contributed to search results. The measurement property searches were not restricted to an older adult population to permit a complete overview of measurement properties of each assessment. In this phase, a study was included if: 1) it was published in English and available in full text, 2) the study included the named assessment and had not modified the assessment in any way, 3) the study aim was to evaluate the measurement quality of the tool, and 4) it was original research. Studies were excluded if they used a translated version of the assessment. If studies referenced published articles on the initial development of the assessment then these additional studies were included in this phase 2 methodology critique.

#### Methodological quality evaluation of included studies

The methodological quality of included papers was evaluated as described elsewhere [19]. Briefly, one rater (KW) trained in the use of the **CO**nsensus-based **S**tandards for the selection of health **M**easurement **In**struments (COSMIN) tool assessed the quality of all included papers on the COSMIN 4-point modular checklist. A rating of poor, fair, good or excellent awarded for methodological quality is awarded for each measurement property [20]. The COSMIN tool provides a systematic approach to analysing the methodological quality of studies that investigate measurement properties of assessment tools and is concerned with how the study was conducted, for example, hypothesis formed and populations under study clearly defined [21]. Based on the recent work of Dobson et al, the assessment of study sample size was not included as an indicator of methodological quality for individual studies [22]. Rather, total sample size per measurement property was accounted for in the evidence synthesis stage.

#### Evaluation of measurement properties

The Terwee criteria were then used to explore the quality of measurement properties, including content validity, internal consistency, construct validity, reliabilities, floor and ceiling effects, and interpretability [23]. The Terwee criteria provides a benchmark to determine whether results of testing of measurement properties are within an acceptable range [23]. For example, an intra-class correlation coefficient of 0.70 is considered an acceptable value in reliability studies [23]. To align the definition of responsiveness and construct validity between the COSMIN checklist and Terwee criteria, the definition of responsiveness was extended to include the ability of the assessment to detect change over time in the construct being measured, and the definition of construct validity was extended to include discriminate and group discriminate validity [20]. Criterion validity was not assessed as no agreed gold standard of functional ability currently exists. Further, additions to the Terwee criteria included a minimum correlation coefficient of ≥0.40 in construct validity and responsiveness studies and/or 75% of hypothesis proven, and the use of an expert to assess item response theory (IRT) methods for structural validity, similar to Brédart et al. [24]. Measurement properties were rated as positive ‘+’, indeterminate ‘?’, negative ‘-’ or no information ‘0’.

#### Clinical utility

Clinical utility of included assessments was completed using guidelines developed by Streiner and Norman, adapted by Zwakhalen and Hamers [25]. A score of two was awarded if the scale was short, manageable, had instructions and guidelines for the interpretation of scores, one was awarded if the scale was manageable and zero if the scale was more complex (for example, different scoring methods used throughout).

#### Synthesis of best evidence for older adults: Measurement properties

Information on methodological quality and robustness of measurement properties was synthesised to determine best evidence. Only papers reporting on older adult populations were included in the data synthesis stage to provide a clear indication of the tools quality when applied to this group set. Studies of poor methodological quality did not contribute to best evidence and were not synthesised [23]. The possible levels of evidence for a measurement property are described in Table 1. as outlined by Dobson et al., 2012, and adapted from Terwee et al., 2007 [22, 23].

**Table 1:** Synthesis of Best Evidence Criteria.

Level	Rating	Criteria
Strong	+++ or—(total sample size ≥ 100)	Consistent findings in multiple studies of good methodological quality or one study of excellent methodological quality
Moderate	++ or—(total sample size 50–99)	Consistent findings in multiple studies of fair methodological quality or in one study of good methodological quality
Limited	+ or—(total sample size 25–49)	One study of fair quality
Conflicting	±	Conflicting findings
Unknown	? (total sample < 25)	Only studies of poor methodological quality

## Results

### Phase 1: Identification of functional assessments used with older adults

From 56 studies, 28 individual assessments were identified (Table 2). If it was unclear which version of the assessment tool was used in a study, studies were assumed to be using the original version. Concepts measured by each assessment are outlined in Table 2, all of which fell under the activities and participation domains of the ICF.

**Table 2:** Identified Assessments Phase 1.

Assessment (paper identified in phase 1)	Country of development	Items	Subscales	Format	Concept
**AAP** [26]	Australia	21	4	Scale	Participation
ADL Index and IADL Index* [27, 28]	United States of America	12	2	Scale	Activity
**ADL staircase** [29, 30]	Sweden	10	-	Hierarchal scale	Activity
**AMPS** [31, 32]	United States of America	36	2	Scale, transformed to rasch	Activity
**BI (Collin and Wade version)** [33–42]	United Kingdom	10	-	Scale	Activity
**CAFU** [43, 44]	United States of America	15	2	Scale	Activity
**COPM** [45]	Canada	Interview	2	1–10 ‘scale	Activity and Participation
**DRI** [46, 47]	Sweden	12	3	Scale	Activity
**FAI** [41, 48–51]	United Kingdom	15	3	Scale	Participation
**FIM**^**TM**^ [31, 51–54]	United States of America	18	2	Scale	Activity
**FSQ** [55, 56]	United States of America	34	6	Scale	Activity
**GARS** [50, 57]	Netherlands	18	2	Scale	Activity
**HAQ-DI** [58]	United States of America	20	-	Scale	Activity
**IAM** [51]	Sweden	7	-	Scale	Activity
**IDDD** [32]	Netherlands	33		Scale	Activity
**Katz ADL** [59]	United States of America	6	-	Hierarchal scale	Activity
**KB ADL** [46]	United States of America	170	-	2-point scale (achieved, not achieved)	
**Lawton IADL** [60, 61]		8	-	Scale	Activity
**Lifespace assessment mobility** [62]	United States of America	15	-	Yes/no; Frequency	Activity
**Mobility–H** [63]	Denmark	5	-	Yes/no	Activity
**MBI** [26, 64–66]	Australia	10	-	Scale	Activity
**Northwick Park Index of Independence in ADL** [67]	United Kingdom	17	-	Scale	Activity
**NEADL** [33–39, 68–71]	United Kingdom	22	4	Scale	Activity
**OBI** [49, 60, 68, 72–80]	United States of America	10	-	Scale	Activity
**RNLI** [54]	Canada	11	2	Visual Analogue Scale	Activity
**Rivermead ADL** [42]	United Kingdom	31	3	Scale	Participation
**SMAF** [81]	Canada	29	5	Scale	Activity
**Sunnaas ADL** [75]	Norway	12	3	Scale	Activity

AAP, Adelaide Activities Profile; ADL Index and IADL Index, Activities of Daily Living Index and Instrumental Activities of Daily Living Index; ADL Staircase, Activities of Daily Living Staircase; AMPS, Assessment of Motor and Process Skills; BI, Barthel Index; CAFU, Caregiver Assessment of Function and Upset; COPM, Canadian Occupational Performance Measure; DRI, Disability Rating Index; FAI, Frenchay Activity Index; FIM^TM^, Functional Independence Measure; FSQ Functional Status Questionnaire; GARS, Groningen Activity Restriction Scale; HAQ-DI Health Assessment Questionnaire–Disability Index; IAM, Instrumental Activity Measure; IDD, Interview of Deterioration in Daily Activities in Dementia; Katz ADL, Katz Activities of Daily Living; KB ADL, Klein Bell Activities of Daily Living; Lawton IADL, Lawton Instrumental Activities of Daily Living; MBI, Modified Barthel Index; Northwick Park Index of Independence in ADL, Northwick Park Index of Independence in Activities of Daily Living; NEADL, Nottingham Extended Activities of Daily Living; OBI, Original Barthel Index; RNLI, Reintegration to Normal Living Index; Rivermead ADL, Rivermead Activities of Daily Living assessment; SMAF, Functional Autonomy Measurement System; Sunnaas ADL, Sunnaas Activities of Daily Living Index.

### Phase 2: Measurement properties of identified functional assessments

Due to the volume of data, detailed information is presented on the ratings for the COSMIN criteria in supplemental documentation (S2 Information) and for Terwee criteria (S3 Information).

### Synthesis of best evidence: measurement properties

Synthesis of best evidence was restricted to studies with older adult populations to enable a review of measurement properties specific to this group set. The number of studies evaluating each assessment tool varied considerably. The BI (C&W) had the highest number of studies with eight contributing to the best evidence synthesis whereas only one was located for the NEADL assessment. No studies were located that were conducted with English speaking populations for the following assessments; the ADL staircase, DRI, GARS, IAM, IDD, Mob-H and Sunnaas ADL assessment. This was expected as these tools were developed in non-English speaking countries. See Table 3 for results.

**Table 3:** Synthesis of best evidence for older adult group set.

Tool	Internal consistency	Reliability (Intra)	Reliability (Inter)	Reliability (Retest)	Measurement error	Content validity	Structural validity	Construct validity	Responsiveness	Interpretability	Floor and ceiling effects	Clinical utility
**SMAF**	0	0	++	0	+	0	0	+	+	+++	0	1
**CAFU**	++	0	0	0	0	0	++	++	0	0	0	1
**AMPS**	0	0	0	?	?	0	0	++	++	0	0	0
**FAI**	0	0	?	0	+	0	0	++	0	0	0	1
**FIM**^**TM**^	+++	0	+++	?	0	0	+++	++	—	0	—-	1
**FSQ**	+	0	0	0	0	0	0	±	0	0	-	1
**BI (Collin and Wade)**	0	0	?	0	+	0	?	±	±	0	—	2
**MBI**	-	0	0	?	0	0	-	?	0	0	-	2
**OBI**	-	0	0	?	0	0	-	?	0	0	-	1
**AAP**	—	0	0	0	0	0	—	++	0	0	0	1
**NEADL**	0	0	0	0	0	0	-	?	0	0	0	2
**COPM**	0	0	0	0	0	0	0	?	?	0	0	1
**ADL Staircase**	0	0	0	0	0	0	0	0	0	0	0	1
**DRI**	0	0	0	0	0	0	0	0	0	0	0	1
**GARS**	0	0	0	0	0	0	0	0	0	0	0	2
**HAQ-DI**	0	0	0	0	0	0	0	0	0	0	0	1
**IAM**	0	0	0	0	0	0	0	0	0	0	0	1
**IDDD**	0	0	0	0	0	0	0	0	0	0	0	1
**Katz ADL**	0	0	0	0	0	0	0	0	0	0	0	1
**KB ADL**	0	0	0	0	0	0	0	0	0	0	0	0
**Lawton IADL**	0	0	0	0	0	0	0	0	0	0	0	1
**Lifespace Assessment**	0	0	0	0	0	0	0	0	0	0	0	1
**Mobility-Help**	0	0	0	0	0	0	0	0	0	0	0	1
**Northwick Park ADL**	0	0	0	0	0	0	0	0	0	0	0	1
**Rivermead ADL**	0	0	0	0	0	0	0	0	0	0	0	1
**RNLI**	0	0	0	0	0	0	0	0	0	0	0	1
**Sunnaas ADL**	0	0	0	0	0	0	0	0	0	0	0	1

AAP, Adelaide Activities Profile; ADL Index and IADL Index, Activities of Daily Living Index and Instrumental Activities of Daily Living Index; ADL Staircase, Activities of Daily Living Staircase; AMPS, Assessment of Motor and Process Skills; BI, Barthel Index; CAFU, Caregiver Assessment of Function and Upset; COPM, Canadian Occupational Performance Measure; DRI, Disability Rating Index; FAI, Frenchay Activity Index; FIM^TM^, Functional Independence Measure; FSQ Functional Status Questionnaire; GARS, Groningen Activity Restriction Scale; HAQ-DI Health Assessment Questionnaire–Disability Index; IAM, Instrumental Activity Measure; IDD, Interview of Deterioration in Daily Activities in Dementia; Katz ADL, Katz Activities of Daily Living; KB ADL, Klein Bell Activities of Daily Living; Lawton IADL, Lawton Instrumental Activities of Daily Living; MBI, Modified Barthel Index; Northwick Park Index of Independence in ADL, Northwick Park Index of Independence in Activities of Daily Living; NEADL, Nottingham Extended Activities of Daily Living; OBI, Original Barthel Index; RNLI, Reintegration to Normal Living Index; Rivermead ADL, Rivermead Activities of Daily Living assessment; SMAF, Functional Autonomy Measurement System; Sunnaas ADL, Sunnaas Activities of Daily Living Index.

+++, strong evidence; ++, moderate evidence; +, limited evidence; ---, strong evidence; --, moderate evidence; -, limited evidence; ±, conflicting;?, poor methodological quality, 0, None identified.

In reviewing the measurement properties of these assessments it became clear that not all assessments had been tested in an older adult population. S2 Information (see hyperlinks at end of paper) describes the mean age of all papers for adults and older adults which we assessed using COSMIN and Terwee criteria.

The majority of the studies that investigated the measurement properties of the SMAF were conducted with an older adult population [82–84] and drawn from rehabilitation, community or hospital settings [82–86]. The methodological quality of the studies for all age groups on the SMAF ranged from fair to good. Reasons for such ratings included no description of how missing items were handled during analysis, no evidence that participants functional ability had not changed in reliability studies, no formalised hypothesis for expected results in construct validity and the use of less optimal statistics in responsiveness studies (such as effect sizes) [82, 83]. Four papers were assessed for measurement quality (S3 Information) with three having an older adult population and thus contributing to synthesis for best evidence [82–84]. Synthesis of best evidence for the older adult group set established that the SMAF assessment was found to be related to the amount of nursing care time an individual would require and thus a reflection of the patients functional ability (r = 0.58–0.89, p<0.0001) [83]. The SMAF was also sensitive to change in a geriatric rehabilitation unit (GRU) and day hospital (DH) (SRM and Guyatt Effect sizes (GRU 0.97–2.17; DH 0.29–0.54) [82]. A minimal detectable change of 5 was established for the SMAF for older adults therefore providing a clear indication of when true change was likely to have occurred rather than systematic or random scoring error [84]. The SMAF was found to be a reliable tool amongst assessors (weighted *k* = 0.75) [83] and to have a manageable scoring format with clear instructions for clinical utility [84]. Overall the synthesis of best evidence with older adults has shown that the SMAF had consistent, positive, measurement properties, supporting its use in this population.

The methodological quality of the one study included for the CAFU was of high standard with a rating of ‘good’. This study was conducted with older adult care recipients and therefore was included in the synthesis of best evidence. The paper did not achieve an ‘excellent’ rating as there was a lack of discussion regarding missing items (S2 Information). The CAFU was established to have moderate evidence for both internal consistency (Cronbach’s α = 0.83–0.91), structural validity and construct validity (e.g. CAFU and MMSE r_s_ = -0.48–0.45) [44]. The tool was rated to have a manageable scoring format but had limited information on interpretation of the score.

There were similar reasons for the awarding of methodological quality scores for the studies for all age groups evaluating the measurement properties of the AMPS. In addition to those previously discussed, poor scores for methodological quality were awarded due to the use of untested comparator assessments and/or no description of constructs assessed by comparator assessments and scoring of the AMPS from videotaping [87, 88]. Twenty-two papers were assessed for measurement quality for all age groups, with only four of these having a population of 70 years and older and thus contributing to best evidence. Results of data synthesis for the older adult group set demonstrated that the AMPS motor and FIM^TM^ motor scores were concurrently valid (r = 0.54, r = 0.62 p < .001) along with AMPS process and FIM^TM^ cognitive ratings (r = 0.56, r = 0.62 p<0.001), Further, AMPS process and Older Americans Resources and Services (OARS) ĸ = 0.36 showed moderate agreement in a study of older adults, further contributing to the construct validity of the process subscale [89]. The AMPS motor and process scores were found to be responsive, with the AMPS process score being more responsive than the FIM^TM^ cognitive score in inpatient rehabilitation for older adults [90]. Little information was found on the reliability of the AMPS assessment tool with only studies of poor methodological quality sourced for test-retest reliability and the tools ability to measure true change (otherwise referred to as measurement error) [91]. Synthesis of this information concluded that the AMPS had moderate evidence to support construct validity and responsiveness in an older adult population and no evidence supporting the reliability of the tool. The AMPS was found to have a complex scoring system which may limit its uptake in clinical or hospital settings.

Studies of the FAI assessment in all ages were deemed to be of poor to good methodological quality. In line with other studies, studies reporting on construct validity were rated fair when hypothesis were not set a-priori and when constructs or measurement properties of comparator assessments were not described. Poor methodological quality was also awarded in a study of content analysis as the suitability of the FAI items reflecting the construct was not comprehensively assessed (S2 Information). Seven papers were assessed for measurement quality using the Terwee criteria (S3 Information). Only two papers had an older adult population and thus contributed to the synthesis of best evidence. Results of this synthesis indicated that for the older adult group set, the FAI was shown to have fair evidence for measurement error in an older population [92], and moderate evidence for construct validity with strong relationships with the BI (C&W) and NEADL assessment tools (r_s_ = 0.8–0.90) [93].

Methodological quality for all studies across all age groups on the FIM ranged from poor to excellent. Reasons for scores to receive poor ratings included the use of telephone follow up [94], use of brief version of the FIM [95] and omission of any assessment of uni-dimensionality prior to internal consistency analysis [96]. Twenty-one papers were assessed for measurement quality using the Terwee criteria (S3 Information) with only four having an older adult population and thus contributing to the synthesis of best evidence. The Fioravanti paper, identified in the AMPS searches, was also included [90]. Results of data synthesis for this group set demonstrated that the FIM can discriminate between people residing in a skilled nursing facility, sheltered care and those living independently in the community (FIM Motor F(2,46) = 34.71 *p*<0.05; FIM cognitive F(2,46) = 12.42 *p*<0.05), and was concurrently valid with the AMPS assessment (AMPS motor and FIM motor r = 0.54, AMPS process and FIM cognitive r = 0.56 (*p*<0.001). Structure of the FIM (motor and cognitive subscales) was supported when applied to an older adult population which supports the use of the FIM^TM^ to measure function. Inter-rater reliability was established in one study of older adults, ICC 0.872 (95% CI 0.822–0.908). The FIM demonstrated significant floor and ceiling effects in sphincter management, mobility dimensions and executive functioning skills indicating limitations of the tool more broadly [97].

Studies reporting on the measurement properties of the FSQ, for all age groups, were rated as poor to excellent for methodological quality. Reasons for the awarding of poor methodological quality included uni-dimensionality not assessed in internal consistency studies and no assessment that the construct is relevant to study population and that assessment items comprehensively measure the same construct in content analysis (S2 Information). Two out of the six papers assessed were carried out with an older adult population and therefore contributed to the synthesis of best evidence. The synthesis results for the older adult group set established that the FSQ BADL and intermediate ADL subscales were internally consistent (Cronbachs α = 0.80 and 0.81 respectively) when applied to a community sample of older adults [98]. The FSQ BADL and intermediate ADL scales were concurrently valid with tools measuring similar constructs such as the SF-36 physical functioning subscale (r = 0.51 and 0.76 respectively) [98] and was established to be responsiveness to change over time (e.g. basic ADL SRM = 1.10 and intermediate ADL = 0.89) when applied in a population post-hip replacement. However, the FSQ was identified to have a ceiling effect when applied in an older adult population [98]. Best evidence synthesis of the FSQ established fair evidence for internal consistency and floor and ceiling effects, and conflicting evidence for construct validity when evaluated in older adult populations. The FSQ was found to have a manageable scoring format and provides scores which indicates an individual may be at risk of functional decline. The floor and ceiling effects questions the usefulness of the FSQ for assessing functional ability in older adult populations.

Studies which investigated the measurement properties of the BI often did not clearly identify which version of the tool they used. Methodological quality ranged from poor to good for studies of all age groups. Poor methodological quality was awarded when there was no discussion of constructs measured by comparator assessments meaning the suitability of concurrent validation could not be determined (e.g. [99]), and when modified scoring guidelines were applied (e.g. [100]). The use of percentage agreement rather than the more robust Kappa analysis also resulted in a poor score in reliability studies, along with no time frame provided in a study of test-retest reliability [101]. Refer to S2 Information for further detail regarding each papers methodological quality. The measurement properties of the BI and MBI were evaluated in two papers with older adult populations while the BI(C&W) was assessed in eight (S3 Information). Results from data synthesis of the older adult group set established that all versions of the BI had ceiling effects, potentially resulting in inaccurate reflections of patient ability [93, 102, 103]. The MBI and OBI also demonstrated multi-dimensionality, indicating that the total score may not be truly indicative of functional ability [102]. Construct validity was supported for the BI (C&W) when applied to older adults with correlations established with other assessments of function such as the FAI (r_s_ = 0.826)_._ Construct validity for the OBI and MBI was not established with only one study of poor methodological quality with older adults [95]. The BI (C&W) was the only version to be evaluated for responsiveness in an older adult population with findings producing conflicting results about the tools ability to detect change over time [103, 104]. Synthesis of this information illustrated that there was fair evidence showing that the MBI and OBI were not internally consistent and did not form a unidimensional scale, fair evidence was established for construct validity and that the tool was free of measurement error for the BI (C&W). There was conflicting evidence regarding the responsiveness of the BI (C&W) and fair to moderate evidence that all versions are susceptible to ceiling effects in older adults.

All studies located on the APP were carried out with an older adult population. The AAP had similar reasons for methodological quality scoring as previously discussed, including the lack of clear hypotheses for construct validation (S2 Information). Results of the Terwee analysis (S3 Information) indicated that the AAP was shown to discriminate between domestic health, social circumstances and those receiving formal services [105, 106] and also shown to share relationships with cognitive based assessments, indicating that poorer results of the AAP may be indicative of decreased cognitive abilities [107]. The AAP was internally consistent for only two of the four subscales (domestic chores θ = 0.80, household = 0.70) with two subscales falling outside the acceptable range (θ = 0.52 service to others, θ = 0.51 social activities) and <50% of the variance was accounted for [106]. All studies of the AAP were conducted in an older adult population and it was rated as having a manageable scoring format. Results of synthesis indicated moderate evidence for construct validity and moderate evidence that structural validity and for two subscales internal consistency was weak.

The NEADL was consistently tested in stroke populations and was found to be straightforward to apply and score. Thirteen papers were assessed for methodological and measurement quality (S2 and S3 Informations). Only two studies evaluated the NEADL in an older adult population with results indicating that the NEADL does not conform to the principles of Guttmann scaling (triangular pattern where respondents agreeing to one response are likely to agree with others in the pattern) [108]. When the NEADL was applied to an older adult population with Chronic airway limitation the tool was able to discriminate between those with and without the condition [101]. Synthesis of best evidence showed fair evidence that the structure of the NEADL should be re-evaluated and fair evidence supporting the ability of the NEADL to measure function in an older adult population. Further research is required to clarify the measurement properties of the NEADL in an older adult population.

The following assessments were only evaluated in younger populations (mean age <70 years) (HAQ-DI, Rivermead ADL, Lawton IADL, Lifespace assessment), the population mean age was not specified (Katz, KB-ADL), or only had studies of poor methodological quality conducted with older adults (COPM). The COPM was evaluated in one study with a population of 70 years and over, however, due to inability to ascertain if hypothesis were made a-piori, therefore making interpretation of the findings of the study inconclusive, the study was rated as having poor methodological quality and therefore did not contribute to best evidence. Results regarding the measurement properties of these assessments are described supplementary data (S2, S3, S4 and S5 Informations). Further research regarding the suitability of these assessments for use with populations 70 and years and older is required.

### Phase 2: Recommendation of an assessment tool for use with older adults

Determining which assessment tool is most suitable for the use with older adults was difficult given the varying number of studies undertaken to evaluate each tool and only a handful actually evaluated in an older adult population. To allow for recommendations to be made about the use of assessments with older adults, tools that were found to have unfavourable findings, such as floor and ceiling effects, and/or were not evaluated in an older adult population were ranked lower in Table 3. Tools that were valid and responsive were ranked higher as these criteria were considered essential for the accurate assessment and ongoing evaluation of an older adult’s functional ability.

The SMAF, CAFU, AMPS and FAI were the only assessments where flaws in measurement properties were not identified. The SMAF had consistent measurement properties and was found to be valid and responsive in an older adult population. The FIM, FSQ, BI(C&W) were found to have floor and ceiling effects when applied in an older adult population.

## Discussion

The use of functional assessments with older adults is inconsistent in RCT’s and this is similar to the findings of Buurman and colleagues [12]. This current research has highlighted that even though the 28 functional assessments identified were used with older adults in RCTs, not all have been tested for use in this population, which may lead to erroneous conclusions about the patient’s functional ability.

Assessments were often found to have been modified to suit the needs of the clinical environment or to shorten the tool, but the actual modifications were poorly reported in the papers. For example, there were three versions of the Barthel Index (BI) located in this review but the version of BI used was often not clear. As a result, we recommend that people should refer to the Mahoney Barthel Index [109] as the Original Barthel Index (OBI), the Collin and Wade Barthel Index [110] as BI (Collin and Wade) and the Shah version [111] as Modified Barthel Index in line with publications to date. In future, health professionals and researchers should be explicit in the assessment they have used, referencing the authors of the original or modified tool, and any modifications made. Once the assessment is modified, the assessment can no longer be assumed to hold equivalent measurement properties to the original and thus, should not be referred to by the same name. Further validity and reliability testing would also be required to ensure the modified assessment is psychometrically sound.

There are currently no functional assessments to evaluate older adults which fulfil every criterion considered as being ‘essential measurement properties’ as outlined in the COSMIN and Terwee criteria. The lack of information regarding the assessment tools measurement properties signals the need for further research including validation in older populations. Furthermore, the similar methodological flaws across included studies (e.g. lack of hypothesis formed a-priori in construct validation and no testing of factors prior to internal consistency analysis) highlight the need for particular attention to be made to the methods of psychometric studies. This is similar to the finding of Vergauwen and Huijnen [112], in a review of assessments for people with Chronic Fatigue Syndrome [113].

The results of this current research support the SMAF as a suitable assessment for use with older adults with moderate construct validity and responsiveness and a clear minimal detectable change of five, being defined. The SMAF focuses on activity limitation experienced in everyday activities, mobility, mental functions and communication. Any limitations in activities/ abilities are then considered in reference to available resources to determine if any handicap exists. Assessment is based on what the subject does rather than what the subject could do with ratings scored on a five point scale from independent (0) to complete help (-3) [114]. While the SMAF is a promising assessment for use with older adults, further research is required to confirm that the assessment is internally consistent, reliable when assessments are repeated, content valid and free of floor and ceiling effects. Another assessment that may be suitable for use with older adults, especially as an outcome measure in community populations of older people, is the AMPS with moderate evidence for construct validity and responsiveness to change. Future research should examine the applicability of assessments to different clinical settings and cultural settings to further contribute to the evidence regarding assessment tool suitability.

While this study has demonstrated some important psychometric strengths of these assessment tools, many have limited information on a broad number of measurement properties and require further validation. At this time, health professionals will need to compromise on the measurement properties when selecting a functional assessment tool and should be careful to use assessments that have been tested in their specific population and setting. The choice of functional assessment in clinical or research settings may be influenced not only by psychometric quality but environmental restrictions, budgets, purpose of assessment (self-report versus observational) and how well the assessment aligns with the values of the setting [9, 115]. Previous reviews of assessment tool use in occupational therapy and allied health settings have identified the tensions in selecting standardised assessment tool use and the reasoning for using non-standardised assessments including that they are person centred and easily adaptable (e.g., [115]).

This current study used the ICF definition of function to allow for the occupational therapy role to be described through a common language. We acknowledge that specific occupational therapy models may reflect on aspects of function, in particular delineating activity and participation constructs, in more detail than the ICF [18].

### Limitations

We believe the COSMIN and Terwee methods for health status measures, to be the most robust method to assess methodological quality of studies reporting measurement properties of functional assessments, but acknowledge that further development of these processes may be required in some areas. At times, the COSMIN criteria were somewhat difficult to interpret and consultation with the original developers was required, perhaps making exact replication of our study challenging. This research has formed a basis for further comparisons of functional assessments, including those assessments that we were not able to be included in our review as they were either not published as an outcome measure in a randomised trials or had not been administered on an older population.

We acknowledge that searches conducted were completed in 2012 and that further research regarding measurement property quality may have been conducted since this time. This paper is the first to systematically review the measurement properties of functional assessments used by occupational therapists with an older adult group set. We believe this research can be further contributed to as new research emerges on psychometric quality of functional assessment tools. Additionally, research may be conducted on assessments that were not identified in our review of RCT’s and compared to our results.

## Conclusion

Health professionals and researchers can use the best evidence synthesis to make decisions on the most useful and valid assessment tool for their purposes and settings. Modifications to assessments should be avoided unless the new version is tested appropriately and renamed. Overall, within the criteria set for our study, the SMAF had the most promising psychometric evidence for use with older adults. Further standardisation and evaluation of measurement properties of existing assessments should be undertaken especially in relation to responsiveness which was rarely investigated.

## Supporting Information

S1 Information Example search strategy used in medline (phase two). (DOCX)

S2 Information Methodological quality of measurement properties (COSMIN analysis). (DOCX)

S3 Information Quality of measurement properties (Terwee analysis). (DOCX)

S4 Information Data extracted on reliability. (DOCX)

S5 Information Data extracted on validity. (DOCX)

S6 Information Complete reference list. (DOCX)

S7 Information Prisma Checklist. (DOC)
