# Supplementary material for: Functional Assessments Used by Occupational Therapists with Older Adults at Risk of Activity and Participation Limitations: A Systematic Review
Source: PLoS One. 2016 Feb 9;11(2):e0147980. doi: 10.1371/journal.pone.0147980 (PMC4747506; doi:10.1371/journal.pone.0147980)
Supplement: S2 Information — (DOCX) [file pone.0147980.s002.docx]

**S2 Supporting Information. Methodological quality of measurement properties (COSMIN analysis).**

| **Tool** | **Reference** | **Age**  **Mean (SD)** | **Population**  **n (% men)** | **Diagnostic group** | **Setting** | **Methodological quality** |
| --- | --- | --- | --- | --- | --- | --- |
| AAP | Bond & Clark (1998) [105] | 78.3 (5.9) | n=1799  (54.1%) | Community dwelling | Community | Construct validity (f) |
|  | Clark & Bond (1995) [106] | 78 (mean) (range 70-103) | n=1799  (54.1%) | Community dwelling | Community | Internal Consistency (g)  Construct validity (f)  Structural validity (g) |
|  | Newson & Kemps (2005) [107] | 77.74 (5.51) | n=755  (42.6%) | Community dwelling | Community | Construct validity (f) |
| AMPS | Albert et al., (2006) [87] | >70 | n=188  (30.3%) | Mixed | Community | Construct validity (p) |
|  | Bernspang & Fisher (1995) [116] | Right side stroke=  73.5 (7.8); Left side stroke= 73.4 (7.5), non-disabled 72.9 (7.6) | n=230  (49.1%) | Stroke | AMPS Database | Construct validity (g) |
|  | Doble et al., (1999) [89] | 76.8 (6.6) | n=26  (27%) | Dementia | Community | Construct validity (f) |
|  | Doble, Fisk et al., (1999) [91] | 77.9 (7) | n=55  (33%) | Dementia | Community | Measurement error (p)  Test- retest reliability (p) |
|  | Doble et al., (1997) [88] | Dementia group= 76.5 (8.08); Non dementia group= 75.9 (6.7) | n=64  (39%) | Dementia | Community | Construct validity (p) |
|  | Duran & Fisher (1996) [117] | Men motor= 60.2 (18.0), Females motor = 64.4 (17.8), Men process= 59.8 (18.2) and Females process=64.4 (17.7) | n=3565 motor sample, n=3676 process sample  (39%) | Mixed | AMPS Database | Structural validity (f)  IRT(g) |
|  | Fioravanti et al., (2012) [90] | 80 (8.6) | n=54  (not reported) | Mixed | Rehabilitation | Construct validity (f)  Responsiveness (g) |
|  | Hartman et al., (1999) [118] | Non disabled=70.5 (5.9), dementia minimal assist= 71.2 (9.7) and 74.5 (8.4) | n=788  (35%) | Dementia | AMPS Database | Construct validity (f) |
|  | Kottorp et al., (2003) [119] | 27.7 (16.5) | n=1724  (52%) | Autism, Cerebral Palsy, Intellectual Disability, Spina bifida, impairments of learning | AMPS Database | Structural validity (p)  IRT (f) |
|  | Kottorp (2008) [120] | 37.2 (13.1) | n=380  (46%) | ID | AMPS Database | Construct validity (f) |
|  | McNulty & Fisher (2001) [121] | 58 (16.05) | n=20  (100%) | Mental health | Psychiatric Unit | Construct validity (f) |
|  | Mercier et al., (2001) [86] | 69.8 (mean) (range 55-79) | n=100  (59%) | Stroke | Acute, long term and geriatric centre | Structural validity (f) |
|  | Merritt & Fisher (2003) [122] | 58.7 | n=18500  (50%) | Mixed | AMPS Database | Structural validity (f)  IRT (g) |
|  | Merritt (2010) [123] | Range 16-103 | n=64446  (not reported) | Mixed | AMPS Database | Structural validity (f)  IRT (g) |
|  | Merritt (2011) [124] | Independent= 55.1 (17.9); Minimal Assistance= 57.6 (20.2) and maximal assistance= 61.7 (20.6) | n=64446  (not reported) | Mixed | AMPS Database | Structural validity (f)  IRT (g) |
|  | Pan & Fisher (1994) [125] | 37.9 (14.9) | n=60  (28%) | Mixed | AMPS database | Construct Validity (f) |
|  | Poole, Atansoff et al., (2006) [126] | Systemic Lupus Erythematosus=43, Healthy controls=48 | n=30  (0%) | Systemic Lupus Erythematosus | Community | Construct Validity (f) |
|  | Robinson & Fisher (1996) [127] | 75.4 (9.56) | n=51  (not reported) | Dementia | Community | Construct Validity (f) |
|  | Robinson & Fisher (1999) [128] | People with dementia =72.3 (6.1) and healthy controls= 73.8 (8.2) | n=29  (not reported) | Dementia | Community | Construct Validity (f) |
|  | Stauffer et al., (2000) [129] | African Americans= 54.7 (18.2) and Caucasian= 54.8 (18.3) | n=466  (not reported) | Mixed | AMPS Database | Structural validity (f) |
|  | White & Mulligan (2005) [130] | Healthy controls= 8.55 ; Attention deficit hyperactivity disorder= 8.2 | n=33  (not reported) | Attention deficit hyperactivity disorder | Community | Construct Validity (f) |
|  | White et al., (2007) [131] | 7.9 (2.25) | n=68  (68%) | Sensory processing difficulties | Community | Construct Validity (f) |
| BI | Ahmed et al., (2003) [132] | 67 (14) | n=63  (62%) | Stroke | Hospital | Construct validity (f)  Responsiveness (f) |
|  | de Morton et al., (2008) [102] | 78.9 (7.5) | n=236  (45%) | Older Adults | Hospital | Internal consistency (g)  Structural validity (g)  IRT (g)  Ceiling effect* |
|  | Filiatrault et al., (1991) [133] | 52.2 (13.5) | n=18  (67%) | Upper Limb difficulties | Rehabilitation | Construct validity (p)  Responsiveness (p) |
|  | Frick & Unsworth (1996) [95] | 75.4 (mean) (range 52-87) | n=25  (48%) | Mixed | Hospital | Test-retest reliability (p) Construct validity (p) |
|  | Khan et al., (2008) [134] | 52 (8.3) | n=24  (42%) | Multiple Sclerosis | Hospital | Responsiveness (g) |
|  | Kwon et al., (2004) [135] | 70 (11.4) | n=1680  (46.6%) | Stroke | Hospital | Construct validity (f) |
|  | Wallace et al., (2002) [136] | 69.7 (11.6) | n=372  (48%) | Stroke | Hospital | Responsiveness (f) |
|  | Wellwood et al., (1995) [137] | 73 (13.4) | n=152  (45%) | Stroke | Community | Construct validity (f)  Ceiling effect |
| BI (Collin and Wade) | Al-Khawaja et al., (1997) [138] | 49 (13) | n=132  43% | Stroke, Multiple Sclerosis, Spinal Cord Injury, neurodegenerative and others | Hospital | Construct validity (f) |
|  | Barer & Murphy (1993) [100] | 73.2 | n=730  46% | Stroke | Hospital | Construct validity (p)  Structural validity (p)  Responsiveness (p) |
|  | Brazil et al., (1997) [139] | Median= 57 (range 23-77) | n=107  (29%)) | Brain tumor | Neuro-oncology unit | Construct validity (p)  Responsiveness (p) |
|  | Dennis et al., (2000) [99] | 64.6 | n=417  (not reported) | Stroke | Mixed | Construct validity (p) |
|  | Green et al., (2001) [140] | 71.6 (6.8) | n=22  73% | Stroke | Community | Test-retest reliability (p)  Measurement error (g) |
|  | Gompertz et al., (1994) [141] | Not Reported | n=191  (not reported) | Stroke | Community | Construct validity (p) |
|  | Hartigan & O’Mahony (2011) [142] | 81.5 (7.46) | n=65  (38%)) | Not reported | Hospital | Inter-rater reliability (p)  Measurement error (g) |
|  | Harwood & Ebrahim (2000) [104] | 83 (mean) (range 68-96) | n=54  (35%) | Not reported | Day Hospital | Construct validity (p)  Responsiveness (f) |
|  | Hatfield et al., (2003) [143] | Group 1 45.33(17.15)  Group 2 41.20 (15.29) | n=20  (Group 1 – 50% males, Group 2, not reported) | Head injury, Cerebral Palsy and post-meningioma, stroke | Hospital | Construct validity (f) |
|  | Hobart eet al., (2001) [96] | 46.2 (14.8) (range 16-77) | n=149  46.6% | Stroke, Multiple Sclerosis and head injury | Hospital | Internal consistency (p)  Construct validity (f)  Responsiveness (f) |
|  | Hobart et al., (2010) [144] | 49 (15) | n=1396  (44%) | Neurological | Hospital | Responsiveness (f)  IRT (f)  Ceiling effect |
|  | Houlden et al., (2006) [145] | TBI=37.5; Vascular=47.7 | n=259  (not reported) | Vascular brain injury | Hospital | Responsiveness (f)  Floor effect |
|  | Kidd et al., (1995) [146] | Not Reported | n=25  (not reported) | Neurological | Hospital | Inter-rater reliability (p)  Measurement error (f)  Construct validity (f)  Responsiveness (f)  IRT (f) |
|  | Parker et al., (1994) [147] | 81 for inpatients (range 75-86) | n=540  (53%) | Mixed | Inpatient and Geriatric Unit | Construct validity (p)  Responsiveness (p) |
|  | Richards et al., (2000) [148] | 79 (median) (range 73-84) | n=94  (not reported) | Not reported | Hospital | Inter-rater reliability (p)  Measurement error (p) |
|  | Sarker et al., (2012). [149] | 68.6 (14.2) | n=238  (52%) | Stroke | Community | Construct validity (p)  Ceiling effect (BI), Floor effect (FAI) |
|  | Van Der Putten et al., (1999) [150] | Multiple Sclerosis 45 (11.2); Stroke 52 (16.9) | n=283  (83.8%) | Multiple Scelrosis & Stroke | Neuro-rehabilitation | Responsiveness (f)  Ceiling effect (FIM cognitive subscale) |
|  | Wade & Collin (1998) [151] | 12-66+ | n=25  (68%) | Stroke | Hospital | Inter-rater reliability (p) |
|  | Wade & Hewer (1987) [152] | Not Reported | n=713  (not reported) | Stroke | Community | Construct validity (f)  Structural validity (g) |
|  | Wilkinson et al., (1997) [93] | 71 (median) (range 34-79) | n=106  (54%) | Stroke | Community | Construct validity (f)  Ceiling effect |
|  | Wright et al., (1998) [103] | 83 (mean) (range 63-99) | n=54  (38%) | Mixed | Hospital | Construct validity (f)  Responsiveness (f)  Ceiling effect |
|  | Yohannes et al., (1997) [101] | 78 (mean) (range 70-93) for chronic airway limitation, 78 (mean) (range 71-90) for control | n=151  (52%) | Chronic airway limitation | Community | Measurement error (p)  Construct validity (f) |
| CAFU | Gitlin et al., (2005) [44] | Caregiver 61.9 (13.5) Care recipient age 79.3 (7.9) | n=640  (39%) | Alzheimer’s Disease | Community | Internal consistency (g)  Construct validity (g)  Structural validity (g) |
| COPM | Bodiam (1999) [153] | 52 (mean) (range 17-69) | n=17  59% | Neurological | Neurorehabilitation | Construct validity (f)  Responsiveness (f) |
|  | Carpenter et al., (2001) [154] | 44 (mean) (range 19-72) | n=87  (45%) | Pain | Community | Construct validity (f)  Responsiveness (f) |
|  | Case-Smith (2003) [155] | 44 | n=33  (39%) | Hand treatment | Outpatients | Construct validity (f)  Responsiveness (f) |
|  | Chan & Lee (1997) [156] | 64.5 | N=39  (28%) | Orthopedic and stroke | Rehabilitation | Content validity (g)  Construct validity (f) |
|  | Donnelly et al., (2004) [157] | 49 (18.1) | n=41  (76%) | SCI | Rehabilitation | Construct validity (f)  Responsiveness (f) |
|  | Edwards et al., (2007) [158] | 80.8 (7.3) | n=50  (24%) | Hip fracture | Rehabilitation | Construct validity (p)  Responsiveness (p) |
|  | Jenkinson et al., (2007) [159] | 44.59 (12.34) | n=34  (53%) | Acquired Brain Injury | Rehabilitation | Test-retest reliability (g)  Construct validity (f)  Responsiveness (f) |
|  | McColl et al., (2000) [160] | 50%>65 | n=62  (40%) | Community | Community | Construct validity (g) |
|  | Law et al., (1994) [161] | Not reported | n=256  (42.2%) | Mixed | Mixed | Construct validity (f)  Responsiveness (f) |
|  | Ripat et al., (2001) [162] | Not reported | n=13  (15%) | Rheumatoid Arthritis | Community | Construct validity (f) |
|  | Rochman et al., (2008) [163] | 40 (mean) (range 22-50) | n=29  (21%) | Pain | Pain centres | Construct validity (f)  Responsiveness (f) |
|  | Sewell & Singh (2001) [164] | 67.1 (7.4) | n=15  (not reported) | Chronic Obstructive Pulmonary Disease | Outpatients | Test-retest (g)  Measurement error (g) |
|  | Stuber & Nelson (2010) [165] | 74.0 (9.9) | n=30  (40%) | Orthopaedic, cardiovascular, respiratory, others | Hospital | Construct validity (g) |
|  | van Huet & Williams (2007) [166] | 46 | n=64  (44%) | Pain | Community | Construct validity (f)  Responsiveness (p) |
|  | Walsh et al., (2004) [167] | 46 (median) (Inter-quartile range 39-54) | 101  (51%) | Pain | Pain management program | Construct validity (f)  Responsiveness (f) |
| FAI | Green et al., (2001) [140] | 71.6 (6.8) | n=22  (73%) | Post-stroke | Community | Test-retest reliability (p)  Measurement error (f) |
|  | Carter et al., (1997) [92] | 71 (mean) (range 46-88) | n=48  (57%) | Stroke | Community | Inter-rater reliability (p) Measurement error (g)  Construct validity (f) |
|  | Holbrook & Skilbeck (1983) [168] | Not reported | n=122  (53%) | Stroke | Community | Construct validity (p)  Structural validity (g) |
|  | Patel et al., (2006) [169] | 68.6 (13.5) | n=490  (53.9%) | Stroke | Community | Construct validity (f) |
|  | Sarker et al., (2012) [149] | 68.6 (14.2) | n=238  (52%) | Stroke | Community | Construct validity (p)  Floor effect |
|  | Turnbull et al., (2000) [170] | Median age of men 60 (Inter-quartile range 40.5-77) and women 55 (Inter-quartile range 35-71) | n=602  (47%) | Not reported | Community | Test-retest reliability (g) Content validity (p)  Construct validity (p)  Ceiling effect (domestic subscale) |
|  | Wilkinson et al., (1997) [93] | 71 (34-79) | n=106  (54%) | Stroke | Community | Construct validity (f) |
| FIM | Corrigan et al., (1997) [171] | 35.2 | n=95  (69.5%) | Traumatic Brain Injury | Community | Construct validity (f)  IRT (f) |
|  | Dodds et al., (1993) [172] | 65 | n=11,102  (51%) | Mixed | Rehabilitation | Internal consistency (g)  Construct validity (e)  Responsiveness (f) |
|  | Fricke & Unsworth (1996) [95] | 75.4 (range 52-87) | n=25  (48%) | Mixed | Hospital | Construct validity (p) |
|  | Glenny et al., (2010) [173] | 78.5 (9.3) | n=208  (33%) | Mixed | Rehabilitation | Responsiveness (p) |
|  | Graves (2005) [174] | Not reported | n=440  (not reported) | Spinal Cord Injury | Hospital | Structural Validity (f)  IRT (f) |
|  | Hall et al., (1993) [94] | Not reported | n=332  (not reported) | Traumatic Brain Injury | Database | Construct validity (p)  IRT (f)  Ceiling effect |
|  | Hamilton & Granger (1994) [175] | Not reported | n=1018  (not reported) | Mixed | Rehabilitation | Inter-rater reliability (f) |
|  | Heinemann et al., (1993) [176] | 62.1 | n=27,669  (47%) | Mixed | Mixed | Structural validity (g)  IRT (g) |
|  | Heinemann et al., (1994) [177] | 62.1 | n=27,669  (47%) | Mixed | Mixed | Structural validity(g)  IRT (g) |
|  | Heinemann et al., (1997) [178] | TBI 37.4 (19.5) and SCI 38.9 (19.0) | n=182  (71%) | Traumatic Brain Injury and Spinal Cord Injury | Rehabilitation | Construct validity (p)  IRT (f) |
|  | Hobart et al., (2001) [96] | 46.2 (14.8) (range 16-77) | n=149  (46.6%) | Stroke, Multiple Sclerosis and head injury | Hospital | Internal consistency (p)  Intra-rater reliability (f)  Construct validity (f)  Responsiveness (f) |
|  | Houlden et al., (2006) [145] | Vascular brain injury 48 (10.4), Infarct 48 (11.3), subarachnoid haemorrhage 50 (8.7), intracerebral haemorrhage 45 (9.9), traumatic brain injury 38 (13.4) | n=259  (not reported) | Brain Injury | Neurological rehabilitation | Responsiveness (f) |
|  | Jette et al., (2005) [97] | 76.3 (95% CI 76.1-76.6) | n=7526  (38%) | Mixed | Mixed | Internal consistency (e)  Structural validity (e)  Floor & ceiling effect |
|  | Kidd et al., (1995) [146] | Not Reported | n=25  (not reported) | Neurological | Hospital | Inter-rater reliability (p)  Measurement error (f)  Construct validity (f)  Responsiveness (f)  IRT (f) |
|  | Kohler et al., (2010) [179] | 76 | n=143  (not reported) | Mixed | Rehabilitation | Inter-rater reliability (e) |
|  | Linacre et al., (1994) [180] | Not reported | n=14,799  (not reported) | Mixed | Rehabilitation | Structural validity (g)  IRT (g) |
|  | Ottenbacher et al., (1994) [181] | 75.70 (8.10) | n=20  (50%) | Mixed | Community | Inter-rater reliability (f)  Test-retest reliability (f) |
|  | Pollak et al., (1996) [182] | 89.7 (range 80-104) | n=49  (0.06%) | Mixed | Care facility | Test-retest reliability (p)  Construct validity (f)  Structural validity (g)  IRT (g) |
|  | Segal et al., (1993) [183] | Not reported | n=57  (not reported) | Mixed | Rehabilitation | Inter-rater reliability (p) |
|  | Sharrack et al., (1999) [184] | Median 40 (range 22-74) | n=64  (34%) | Multiple Sclerosis | Hospital | Internal consistency (g)  Inter & intra-rater reliability (g)  Construct validity (g) Structural validity (g)  Responsiveness (f) |
|  | Stineman et al., (1996) [185] | Range 42.1-77.6 | n=84,537  (not reported) | Mixed | Rehabilitation | Internal consistency (g)  Structural validity (g) |
| FSQ | Jette et al., (1986) [186] | Range 19-96 | n=1,153  (25%) | Ambulatory patients | Outpatients clinic | Internal consistency (p)  Content validity (p)  Construct validity (f) |
|  | Katz et al., (1992) [187] | 59.7 (13) | n=54  (33%) | Hip replacement | Hospital | Construct validity (f)  Responsiveness (f) |
|  | Reuben et al., (1992) [188] | 73 (range 64-92) | n=282  (37%) | Community dwelling | Community | Construct validity (f) |
|  | Reuben et al., (1995) [98] | 76 (range 64-92) | n=83  (46%) | Frail older adults | Community | Internal consistency (e)  Construct validity (f)  Ceiling effect |
|  | Rubenstein et al., (1998) [189] | 61 (10.8) | n=193  (58.5%) | Parkinsons disease | Clinic | Internal consistency (p)  Construct validity (g) |
|  | Yarnold et al., (1995) [190] | ≥65 mean 72.6 (6.6)  <65 mean 41.8 (13.0) | n=125  (Group 1 – 17.5% and Group 2 28.2%) | Ambulatory patients | Clinic | Internal consistency (p)  Construct validity (g) |
| HAQ-DI | Benton et al., (2004) [191] | Median 48.5 | n=34  (38%) | Early Rheumatoid Arthritis | Community | Construct validity (f) |
|  | Bombardier & Raboud (1991) [192] | Not provided | n=303  (not reported) | Rheumatoid Arthritis | Community | Measurement error (p)  Responsiveness (f) |
|  | Bruce & Fries (2004) [193] | 65.7 (0.6) | n=271  (21%) | Osteoarthritis | Community | Construct validity (f)  Responsiveness (g) |
|  | Buchbinder et al., (1995) [194] | 53.3 (1.1) | n=77  (31%) | Rheumatoid Arthritis | Clinical Centre | Responsiveness (f) |
|  | Clements et al., (2001) [195] | 43.7 (12.4) | n=134  (not reported) | Scleroderma | Community | Construct validity (g)  Responsiveness (p) |
|  | Cole et al., (2005) [196] | 51 (13) | n=278  (not reported) | Rheumatoid Arthritis | Community | Structural validity (g) |
|  | Cole et al., (2006) [197] | 47.75 (11.28) | n=387  (not reported) | Scleroderma | Community | Structural validity (e) |
|  | Fries & Ramey (1997) [198] | 63 (range 22.6-88.5) | n=663  (18.9%) | Rheumatoid Arthritis | Community | Construct validity (f)  Responsiveness (f) |
|  | Lawrence et al., (2009) [199] | Sample 1 58.47 (1.05) Sample 2 55.04 (0.72) | n=120 sample 1  n=294 sample 2  (not reported) | Rheumatoid Arthritis | Community | Construct validity (p)  Responsiveness (p) |
|  | Leigh & Fries (1992) [200] | 52 (14) | n=209  (14%) | Rheumatoid Arthritis | Community | Construct validity (f) |
|  | Marra, Rashidi et al., (2005) [201] | 61.5 (25.9) | n=239  (not reported) | Rheumatoid Arthritis | Community | Test-retest reliability (f)  Responsiveness (f) |
|  | Marra, Woolcott, et al., (2005) [202] | 61.5 (25.9) | n=313  (22%) | Rheumatoid Arthritis | Community | Construct validity (g) |
|  | Milligan et al., (1993) [203] | 33 (13) | n=130  (0%) | Systematic Lupus Erythematosus | Community | Internal Consistency (g) Construct validity (f) Structural validity (g) |
|  | Poole et al., (1995) [204] | 49.1 (range 23-71) | n=80  (not reported) | Scleroderma | Outpatients | Inter-rater reliability (p) Construct validity (p) |
|  | Poole et al., (2006) [205] | 49.5 (range 22-76) | n=40  (15%) | Rheumatoid Arthritis | Not reported | Construct validity (f) |
|  | Ripat et al., (2001) [162] | Not Reported | n=13  (15%) | Rheumatoid Arthritis | Community | Construct validity (f) |
|  | Rohekar & Pope (2009) [206] | 59.91 (11.83) | n=122  (20%) | Rheumatoid Arthritis | Clinic | Test-retest reliability (g) |
|  | Sultan et al., (2004) [207] | Median 43.5 | n=205  (not reported) | Scleroderma | Not specified | Construct validity (p) |
|  |  |  |  |  |  |  |
| Katz ADL | Katz et al., (1963) [208] | 60% 60 years + | n=1001  (not reported) | Mixed | Hospital | Inter-rater reliability (p)  Construct validity (p)  Structural validity (p) |
|  | Katz et al., (1970) [209] | 75% 65 years + | (not reported) | Mixed | Hospital | Content validity (p)  Construct validity (p) |
| KB ADL | Klein & Bell (1982) [210] | Not Reported | (not reported) | Spinal Cord Injury, Stroke, Traumatic Brain Injury | Hospital | Inter-rater reliability (p) Construct validity (p) |
| Lawton IADL | Lawton & Brody (1969) [211] | >60 | n=265  (not reported) | Mixed | Community | Inter-rater reliability (f)  Construct validity (f)  Structural validity (p) |
| Lifespace assessment (mobility) | Baker et al., (2003) [212] | 75 (6.8) | n=306  (46%) | Community dwelling | Community | Test-retest reliability (g)  Construct validity (p) |
|  | Crowe et al., (2008) [213] | 74 | n=624  (47%) | Community dwelling | Community | Construct validity (g) |
|  | Peel et al., (2005) [214] | 75.3 (6.7) | n=998  (50%) | Community dwelling | Community | Construct validity (f) |
| MBI | de Morton et al., (2008) [102] | 78.9 (7.5) | n=236  (45%) | Older adults | Hospital | Internal consistency (g)  Structural validity (g)  IRT (g)  Ceiling effect |
|  | Fricke & Unsworth (1996) [95] | 75.4 (range 52-87) | n=25  (48%) | Mixed | Hospital | Test-retest reliability (p) Construct validity (p) |
|  | Hocking et al., (1999) [215] | 69.5 | n=100  (54%) | Stroke | Rehabilitation/community | Responsiveness (p) |
|  | Shah et al., (1989) [111] | Not reported | n=258  (not reported) | Stroke | Rehabilitation | Internal consistency (p)  Responsiveness (f) |
|  | Shah et al., (2000) [216] | 28 (10.67) | n=78  (85%) | TBI | Rehabilitation | Construct validity (g) |
|  | Shah & Muncer (2003) [217] | 28 (10.67) | n=78  (85%) | TBI | Rehabilitation | Construct validity (g)  Responsiveness (f)  Floor and ceiling effect |
| NEADL | Ashburn et al., (2008) [218] | Non-repeat faller 69.7 (13.3), Repeat Faller 70.7 (11.0) | n=115  Non repeat faller (69%) and repeat faller (65%) | Post-stroke | Community | Construct validity (g) |
|  | das Nair et al., (2011) [219] | 69.17 (10.78) | n=210  (54.8%) | Post-stroke | Community | Structural Validity (g)  IRT (g) |
|  | Gladman et al., (1993) [108] | Median 71 | n=303  (52%) | Post-stroke | Community | Construct validity (p)  Structural validity (f)  IRT (f) |
|  | Gompertz et al., (1993) [220] | 69 | n=21  (57%) | Post-stroke | Community | Test-retest reliability (g)  Measurement error (g) |
|  | Gompertz et al, (1994) [141] | Not reported | n=191  (not reported) | Post-stroke | Community | Construct validity (p)  Responsiveness (p) |
|  | Green et al., (2001) [140] | 71.6 (6.8) | n=22  (73%) | Post-stroke | Community | Test-retest reliability (p)  Measurement error (f) |
|  | Harwood & Ebhrahim (2000) [104] | 72 (10) | n=81  (32%) | Hip replacement | Community | Responsiveness (f) |
|  | Harwood & Ebrahim (2002) [221] | 72 | n=81  (32%) | Hip replacement | Hospital to community | Internal consistency (g) Test-retest reliability (g)  Structural validity (f)  Responsiveness (f)  IRT (f) |
|  | Jacob-Lloyd et al., (2005) [222] | Not reported | n=55  (56%) | Post-stroke | Hospital to community | Construct validity (f)  Responsiveness (f) |
|  | Lincoln & Gladman (1992) [223] | Group 1 n=49 64 (10) range 21-81; Group 2 n=303 70 (11) 44-100 | Group 1 n=49 (57%), Group 2 n=303 (48%) | Post-stroke | Community | Structural validity (f)  IRT (f) |
|  | Nicholl et al., (20022) [224] | 43.2 (10.8) | n=240  (30%) | Post-stroke | Community | Internal consistency (g)  Test-retest reliability (g)  Construct validity (f)  Structural validity (g)  IRT (f) |
|  | Nouri & Lincoln, (1987) [225] | Not reported | n=80  n=20 for reliability  (not reported) | Post-stroke | Community | Test-retest reliability (p)  Structural validity (f)  IRT (f) |
|  | Sarker et al., (2012) [149] | 68.6 (14.2) | n=238  (52.1%) | Post-stroke | Community | Construct validity (p) |
| RNLI | Wood-Dauphinee et al., (1988) [226] | Not reported | (not reported) | Mixed | Hospital to community | Internal consistency (p)  Inter-rater reliability (f)  Content validity (g)  Construct validity (f)  Structural validity (g)  Responsiveness (f) |
| Rivermead ADL | Lincoln & Edmans (1990) [227] | Range 39-89 | n=150  (not reported) | Stroke | Hospital | Structural validity (f) |
|  | Whiting & Lincoln (1980) [228] | Not reported | Sample 1 n=50, sample 2 n=50 and sample 3 n=15  (not reported) | Stroke | Hospital | Inter-rater reliability (f)  Test-retest reliability (f)  Structural validity (p) |
| SMAF | Demers et al., (2010) [82] | 80 (7) | 237  (33.7%) | Mixed | Rehabilitation | Responsiveness (f) |
|  | Desrosiers et al., (2003) [85] | 69.9 (13.5) | n=132  (52.3%) | Stroke | Rehabilitation | Responsiveness (g)  Construct validity (g) |
|  | Hebert et al., (1997) [84] | 80.0 | n=572  (35%) | Older adults | Community | Measurement error (f) |
|  | Hebert et al., (1988) [83] | ‘Older Adults’ | n=146 (reliability study)  n=99 validity study  (not reported) | Older adults | Community and hospital | Construct validity (f)  Inter-rater reliability (g) |

AAP (Adelaide Activities Profile), AMPS (Assessment of Motor and Process Skills), BI (Barthel Index), CAFU (Caregiver Assessment of Function and Upset), COPM (Canadian Occupational Performance Measure), FAI (Frenchay Activity Index), FIM (Functional Independence Measure), FSQ (Functional Status Questionnaire), HAQ-DI (Health Assessment Questionnaire –Disability Index), Katz ADL (Katz Activities of daily living), KB ADL (Klein Bell Activities of Daily Living), Lawton IADL (Lawton Instrumental Activities of Daily Living), MBI (modified Barthel Index) NEADL (Nottingham Extended Activities of Daily Living), RNLI (Reintegration to Normal Living Index), Rivermead ADL (Rivermead Activities of Daily living assessment), SMAF (Functional Autonomy Measurement System)
