# Supplementary material for: Functional Assessments Used by Occupational Therapists with Older Adults at Risk of Activity and Participation Limitations: A Systematic Review
Source: PLoS One. 2016 Feb 9;11(2):e0147980. doi: 10.1371/journal.pone.0147980 (PMC4747506; doi:10.1371/journal.pone.0147980)
Supplement: S3 Information — (DOCX) [file pone.0147980.s003.docx]

**S3 Supporting Information. Quality of measurement properties (Terwee analysis)*.**

| **Measure**  **(target population)** | **Author (year)** | **Internal Consistency** | **Reliability**  **Intra** | **Reliability**  **(inter)** | **Reliability**  **(retest)** | **Measurement Error** | **Content Validity** | **Structural Validity** | **Construct Validity** | **Responsiveness** | **Interpretability** | **Floor & ceiling effects** |
| --- | --- | --- | --- | --- | --- | --- | --- | --- | --- | --- | --- | --- |
| AAP (Community dwellers) | Bond & Clark (1998)  [105] | 0 | 0 | 0 | 0 | 0 | 0 | 0 | + | 0 | 0 | 0 |
| AAP (community dwellers) | Clark & Bond (1995)  [106] | - | 0 | 0 | 0 | 0 | 0 | - | + | 0 | 0 | 0 |
| AAP (Community dwellers) | Newson & Kemps (2005) [107] | 0 | 0 | 0 | 0 | 0 | 0 | 0 | - | 0 | 0 | 0 |
| AMPS (mixed) | Albert et al., (2006) [87] | 0 | 0 | 0 | 0 | 0 | 0 | 0 | ? | 0 | 0 | 0 |
| AMPS (stroke) | Bernspang & Fisher (1995) [116] | 0 | 0 | 0 | 0 | 0 | 0 | 0 | + | 0 | 0 | 0 |
| AMPS (dementia) | Doble e tl., (1999) [89] | 0 | 0 | 0 | 0 | 0 | 0 | 0 | + | 0 | 0 | 0 |
| AMPS (dementia) | Doble et al., (1999) [91] | 0 | 0 | 0 | ? | ? | 0 | 0 | 0 | 0 | 0 | 0 |
| AMPS (dementia) | Doble et al., (1997) [88] | 0 | 0 | 0 | 0 | 0 | 0 | 0 | ? | 0 | 0 | 0 |
| AMPS (mixed) | Duran & Fisher (1996) [117] | 0 | 0 | 0 | 0 | 0 | 0 | + | 0 | 0 | 0 | 0 |
| AMPS (mixed) | Fiorvanti et al., (2012) [90] | 0 | 0 | 0 | 0 | 0 | 0 | 0 | + (FIM also) | + (FIM also) | 0 | 0 |
| AMPS (dementia) | Hartman et al., (1999) [118] | 0 | 0 | 0 | 0 | 0 | 0 | 0 | + | 0 | 0 | 0 |
| AMPS (autism, Cerebral Palsy, intellectual disability, Spina Bifida, impairments of learning) | Kottorp et al., (2003) [119] | 0 | 0 | 0 | 0 | 0 | 0 | ? | 0 | 0 | 0 | 0 |
| AMPS (intellectual disability) | Kottorp (2008) [120] | 0 | 0 | 0 | 0 | 0 | 0 | 0 | + | 0 | 0 | 0 |
| AMPS (mental health) | McNulty & Fisher (2001) [121] | 0 | 0 | 0 | 0 | 0 | 0 | 0 | + | 0 | 0 | 0 |
| AMPS (stroke) | Mercier et al., (2001) [86] | 0 | 0 | 0 | 0 | 0 | 0 | + (SMAF also) | 0 | 0 | 0 | 0 |
| AMPS (mixed) | Merritt & Fisher (2003) [122] | 0 | 0 | 0 | 0 | 0 | 0 | + | 0 | 0 | 0 | 0 |
| AMPS (mixed) | Merritt (2010) [123] | 0 | 0 | 0 | 0 | 0 | 0 | + | 0 | 0 | 0 | 0 |
| AMPS (mixed) | Merritt (2011) [124] | 0 | 0 | 0 | 0 | 0 | 0 | + | 0 | 0 | 0 | 0 |
| AMPS (mixed) | Pan & Fisher (1994) [125] | 0 | 0 | 0 | 0 | 0 | 0 | + | 0 | 0 | 0 | 0 |
| AMPS (systemic lupus erythematosus) | Poole et al., (2006) [126] | 0 | 0 | 0 | 0 | 0 | 0 | + | 0 | 0 | 0 | 0 |
| AMPS (dementia) | Robinson & Fisher (1996) [127] | 0 | 0 | 0 | 0 | 0 | 0 | 0 | + (FIM also) | 0 | 0 | 0 |
| AMPS (dementia) | Robinson & Fisher (1999) [128] | 0 | 0 | 0 | 0 | 0 | 0 | 0 | + | 0 | 0 | 0 |
| AMPS (mixed) | Stauffer et al., (2000) [129] | 0 | 0 | 0 | 0 | 0 | 0 | + | 0 | 0 | 0 | 0 |
| AMPS (attention deficit hyperactivity disorder) | White & Mulligan (2005) [130] | 0 | 0 | 0 | 0 | 0 | 0 | 0 | + | 0 | 0 | 0 |
| AMPS (sensory processing difficulties) | White et al., (2007) [131] | 0 | 0 | 0 | 0 | 0 | 0 | 0 | + | 0 | 0 | 0 |
| BI (stroke) | Ahmed et al., (2003) [132] | 0 | 0 | 0 | 0 | 0 | 0 | 0 | + | + | 0 | 0 |
| BI (older adults) | de Morton et al., (2008) [102] | - | 0 | 0 | 0 | 0 | 0 | - | 0 | 0 | 0 | - |
| BI (upper limb difficulties) | Filiatrault et al., (1991)  [133] | 0 | 0 | 0 | 0 | 0 | 0 | 0 | ? | ? | 0 | 0 |
| BI (mixed) | Fricke & Unsworth (1996) [95] | 0 | 0 | 0 | ? | 0 | 0 | 0 | ? | 0 | 0 | 0 |
| BI (multiple sclerosis) | Khan et al., (2008) [134] | 0 | 0 | 0 | 0 | 0 | 0 | 0 | 0 | + |  |  |
| BI (stroke) | Kwon et al., (2004) [135] | 0 | 0 | 0 | 0 | 0 | 0 | 0 | + (FIM also) | 0 | 0 | 0 |
| BI (stroke) | Wallace et al., (2002) [136] | 0 | 0 | 0 | 0 | 0 | 0 | 0 | 0 | + | 0 | 0 |
| BI (stroke) | Wellwood et al., (1995) [137] | 0 | 0 | 0 | 0 | 0 | 0 | 0 | + | 0 | 0 | - |
| BI Collin and Wade (stroke) | Al-khawaja et al., (1997) [138] | 0 | 0 | 0 | 0 | 0 | 0 | 0 | + | 0 | 0 | 0 |
| BI Collin and Wade (stroke) | Barer & Murphy (1993) [100] | 0 | 0 | 0 | 0 | 0 | 0 | ? | ? | ? | 0 | 0 |
| BI Collin and Wade (brain tumour) | Brazil et al., (1997) [139] | 0 | 0 | 0 | 0 | 0 | 0 | 0 | ? | ? | 0 | 0 |
| BI Collin and Wade (stroke) | Dennis et al., (2000) [99] | 0 | 0 | 0 | 0 | 0 | 0 | 0 | ? | 0 | 0 | 0 |
| BI Collin and Wade (stroke) | Green et al., (2001) [140] | 0 | 0 | 0 | ? | + | 0 | 0 | 0 | 0 | 0 | 0 |
| BI Collin and Wade (stroke) | Gompertz et al., (1994) [141] | 0 | 0 | 0 | 0 | 0 | 0 | 0 | ? | 0 | 0 | 0 |
| BI Collin and Wade (not reported) | Hartigan & O’Mahony (2011) [142] | 0 | 0 | ? | 0 | + | 0 | 0 | 0 | 0 | 0 | 0 |
| BI Collin and Wade (not reported) | Harwood & Ebrahim (2000) [104] | 0 | 0 | 0 | 0 | 0 | 0 | 0 | ? | - | 0 | 0 |
| BI Collin and Wade (head injury, Cerebral Palsy, post-meningioma, stroke) | Hatfield et al., (2003) [143] | 0 | 0 | 0 | 0 | 0 | 0 | 0 | + | 0 | 0 | 0 |
| BI Collin and Wade (stroke, multiple sclerosis and head injury) | Hobart et al., (2001) [96] | ? | 0 | 0 | 0 | 0 | 0 | 0 | + (FIM also) | + | 0 | 0 |
| BI Collin and Wade (neurological) | Hobart et al., (2010) [144] | 0 | 0 | 0 | 0 | 0 | 0 | 0 | 0 | + | 0 | - |
| BI Collin and Wade (vascular brain injury) | Houlden et al., (2006) [145] | 0 | 0 | 0 | 0 | 0 | 0 | 0 | 0 | + | 0 | - |
| BI Collin and Wade (neurological) | Kidd et al., (1995) [146] | 0 | 0 | ? | 0 | - | 0 | 0 | + | + | 0 | 0 |
| BI Collin and Wade (mixed) | Parker et al., (1994) [147] | 0 | 0 | 0 | 0 | 0 | 0 | 0 | ? | ? | 0 | 0 |
| BI Collin and Wade (not reported) | Richards et al., (2000) [148] | 0 | 0 | ? | 0 | ? | 0 | 0 | 0 | 0 | 0 | 0 |
| BI Collin and Wade (stroke) | Sarker et al., (2012) [149] | 0 | 0 | 0 | 0 | 0 | 0 | 0 | ? | 0 | 0 | - (ceiling BI, Floor FAI) |
| BI Collin and Wade (multiple sclerosis and stroke) | Van Der Putten et al., (1999) [150] | 0 | 0 | 0 | 0 | 0 | 0 | 0 | 0 | + | 0 | - (Ceiling effect FIM also) |
| BI Collin and Wade (stroke) | Wade & Collin (1988) [151] | 0 | 0 | ? | 0 | 0 | 0 | 0 | 0 | 0 | 0 | 0 |
| BI Collin and Wade (stroke) | Wade & Hewer (1987) [152] | 0 | 0 | 0 | 0 | 0 | 0 | + | + | 0 | 0 | 0 |
| BI Collin and Wade (stroke) | Wilkinson et al., (1997) [93] | 0 | 0 | 0 | 0 | 0 | 0 | 0 | + | 0 | 0 | - |
| BI Collin and Wade (mixed) | Wright et al., (1998) [103] | 0 | 0 | 0 | 0 | 0 | 0 | 0 | + | + | 0 | - |
| BI Collin and Wade (chronic airway limitation) | Yohannes et al., (1998) [101] | 0 | 0 | 0 | 0 | ? | 0 | 0 | - (NEADL also) | 0 | 0 | 0 |
| CAFU (Alzheimer’s disease) | Gitlin et al., (2005) [44] | + | 0 | 0 | 0 | 0 | 0 | + | + | 0 | 0 | 0 |
| COPM (Neurological) | Bodiam (1999) [153] | 0 | 0 | 0 | 0 | 0 | 0 | 0 | + | + | 0 | 0 |
| COPM (Pain) | Carpenter et al., (2001) [154] | 0 | 0 | 0 | 0 | 0 | 0 | 0 | - | - | 0 | 0 |
| COPM (hand treatment) | Case-Smith (2003) [155] | 0 | 0 | 0 | 0 | 0 | 0 | 0 | + | + | 0 | 0 |
| COPM (orthopaedic and stroke) | Chan & Lee (1997) [156] | 0 | 0 | 0 | 0 | 0 | + | 0 | - (FIM also) | 0 | 0 | 0 |
| COPM (spinal cord injury) | Donnelly et al., (2004) [157] | 0 | 0 | 0 | 0 | 0 | 0 | 0 | + (FIM also) | - | 0 | 0 |
| COPM (hip fracture) | Edwards et al., (2007) [158] | 0 | 0 | 0 | 0 | 0 | 0 | 0 | ? | ? | 0 | 0 |
| COPM (acquired brain injury) | Jenekinson et al., (2007) [159] | 0 | 0 | 0 | + | 0 | 0 | 0 | - | - | 0 | 0 |
| COPM (community) | McColl et al., (2000) [160] | 0 | 0 | 0 | 0 | 0 | 0 | 0 | + | 0 | 0 | 0 |
| COPM (mixed) | Law et al., (1994) [161] | 0 | 0 | 0 | 0 | 0 | 0 | 0 | + | + | 0 | 0 |
| COPM (rheumatoid arthritis) | Ripat et al., (2001) [162] | 0 | 0 | 0 | 0 | 0 | 0 | 0 | + | 0 | 0 | 0 |
| COPM (pain) | Rochman et al., (2008) [163] | 0 | 0 | 0 | 0 | 0 | 0 | 0 | + | + | 0 | 0 |
| COPM (chronic obstructive pulmonary disease) | Sewell & Singh (2001) [164] | 0 | 0 | 0 | + | + | 0 | 0 | 0 | 0 | 0 | 0 |
| COPM (orthopaedic, cardiovascular, respirator, others) | Stuber & Nelson (2001)) [165] | 0 | 0 | 0 | 0 | 0 | 0 | 0 | + | 0 | 0 | 0 |
| COPM (pain) | van Huet & Williams (2007) [166] | 0 | 0 | 0 | 0 | 0 | 0 | 0 | + | ? | 0 | 0 |
| COPM (pain) | Walsh et al., (2004) [167] | 0 | 0 | 0 | 0 | 0 | 0 | 0 | + | + | 0 | 0 |
| FAI (stroke) | Green et al., (2001) [140] | 0 | 0 | 0 | ? | + | 0 | 0 | 0 | 0 | 0 | 0 |
| FAI (stroke) | Carter et al., (1997) [92] | 0 | 0 | ? | 0 | + | 0 | 0 | + | 0 | 0 | 0 |
| FAI (stroke) | Holbrook & Skillbeck (1983) [168] | 0 | 0 | 0 | 0 | 0 | 0 | + | ? | 0 | 0 | 0 |
| FAI (stroke) | Patel et al., (2006) [169] | 0 | 0 | 0 | 0 | 0 | 0 | 0 | + (BI C&W also) | 0 | 0 | 0 |
| FAI (stroke) | Sarker et al., (2012) [149] | 0 | 0 | 0 | 0 | 0 | 0 | 0 | ? | 0 | 0 | - |
| FAI (not reported) | Turnbull et al., (2000) [170] | 0 | 0 | 0 | + | 0 | ? | ? | 0 | 0 | 0 | - (domestic subscale) |
| FAI (stroke) | Wilkinson et al., (1997) [93] | 0 | 0 | 0 | 0 | 0 | 0 | 0 | + | 0 | 0 | 0 |
| FIM (traumatic brain injury) | Corrigan et al., (1997) [171] | 0 | 0 | 0 | 0 | 0 | 0 | 0 | + | 0 | 0 | 0 |
| FIM (mixed) | Dodds et al., (1993) [172] | + | 0 | 0 | 0 | 0 | 0 | 0 | + | + | 0 | 0 |
| FIM (mixed) | Fricke & Unsworth (1996) [95] | 0 | 0 | 0 | 0 | 0 | 0 | 0 | ? | 0 | 0 | 0 |
| FIM (mixed) | Glenny et al., (2010) [173] | 0 | 0 | 0 | 0 | 0 | 0 | 0 | 0 | ? | 0 | 0 |
| FIM (spinal cord injury) | Graves (2005) [174] | 0 | 0 | 0 | 0 | 0 | 0 | + | 0 | 0 | 0 | 0 |
| FIM (Traumatic Brain Injury) | Hall et al., (1993) [94] | 0 | 0 | 0 | 0 | 0 | 0 | 0 | ? | 0 | 0 | - |
| FIM (mixed) | Hamilton & Granger (1994) [175] | 0 | 0 | + | 0 | 0 | 0 | 0 | 0 | 0 | 0 | 0 |
| FIM (mixed) | Heinemann et al., (1993) [176] | 0 | 0 | 0 | 0 | 0 | 0 | + | 0 | 0 | 0 | 0 |
| FIM (mixed) | Heinemann et al., (1994) [177] | 0 | 0 | 0 | 0 | 0 | 0 | + | 0 | 0 | 0 | 0 |
| FIM (Traumatic Brain Injury and Spinal Cord Injury) | Heinemann et al., (1997) [178] | 0 | 0 | 0 | 0 | 0 | 0 | 0 | ? | 0 | 0 | 0 |
| FIM (stroke, Multiple Sclerosis and head injury) | Hobart et al., (2001) [96] | ? | 0 | 0 | 0 | 0 | 0 | 0 | + | + | 0 | 0 |
| FIM (brain injury) | Houlden et al., (2006) [145] | 0 | 0 | 0 | 0 | 0 | 0 | 0 | 0 | + | 0 | 0 |
| FIM (mixed) | Jette et al., (2005) [97] | + | 0 | 0 | 0 | 0 | 0 | + | 0 | 0 | 0 | - |
| FIM (neurological) | Kidd et al., (1995) [146] | 0 | 0 | ? | 0 | - | 0 | 0 | + | + | 0 | 0 |
| FIM (mixed) | Kohler et al., (2010) [179] | 0 | 0 | + | 0 | 0 | 0 | 0 | 0 | 0 | 0 | 0 |
| FIM (mixed) | Linacre et al., (1994) [180] |  |  |  |  |  |  |  |  |  |  |  |
| FIM (mixed) | Ottenbacher et al., (1994) [181] | 0 | 0 | + | + | 0 | 0 | 0 | 0 | 0 | 0 | 0 |
| FIM (mixed) | Pollak et al., (1996) [182] | 0 | 0 | 0 | ? | 0 | 0 | + | + | 0 | 0 | 0 |
| FIM (mixed) | Segal et al., (1993) [183] | 0 | 0 | ? | 0 | 0 | 0 | 0 | 0 | 0 | 0 | 0 |
| FIM (Multiple Sclerosis) | Sharrack et al., (1999) [184] | + | + | + | 0 | 0 | 0 |  | + (BI C&W also) | - | 0 | 0 |
| FIM (mixed) | Stineman et al., (1996) [185] | + | 0 | 0 | 0 | 0 | 0 | + | 0 | 0 | 0 | 0 |
| FSQ (ambulatory patients) | Jette et al., (1986) [186] | ? | 0 | 0 | 0 | 0 | ? | 0 | + | 0 | 0 | 0 |
| FSQ (hip replacement) | Katz et al., (1992) [187] | 0 | 0 | 0 | 0 | 0 | 0 | 0 | + | + | 0 | 0 |
| FSQ (community dwelling) | Reuben et al., (1992) [188] | 0 | 0 | 0 | 0 | 0 | 0 | 0 | - | 0 | 0 | 0 |
| FSQ (frail older adults) | Reuben et al., (1995) [98] | + | 0 | 0 | 0 | 0 | 0 | 0 | + | 0 | 0 | - |
| FSQ (Parkinsons Disease) | Rubenstein et al., (1998) [189] | ? | 0 | 0 | 0 | 0 | 0 | 0 | + | 0 | 0 | 0 |
| FSQ (ambulatory patients) | Yarnold et al., (1995) [190] | ? | 0 | 0 | 0 | 0 | 0 | 0 | + | 0 | 0 | 0 |
| HAQ-DI (early rheumatoid arthritis) | Benton et al., (2004) [191] | 0 | 0 | 0 | 0 | 0 | 0 | 0 | + | 0 | 0 | 0 |
| HAQ-DI (rheumatoid arthritis) | Bombardier & Raboud (1991) [192] | 0 | 0 | 0 | 0 | ? | 0 | 0 | 0 | + | 0 | 0 |
| HAQ-DI (osteoarthritis) | Bruce & Fries (2004) [193] | 0 | 0 | 0 | 0 | 0 | 0 | 0 | + | + | 0 | 0 |
| HAQ-DI (rheumatoid arthritis) | Buchbinder et al., (1995) [194] | 0 | 0 | 0 | 0 | 0 | 0 | 0 | 0 | + | 0 | 0 |
| HAQ-DI (scleroderma) | Clements et al., (2001) [195] | 0 | 0 | 0 | 0 | 0 | 0 | 0 | + | ? | 0 | 0 |
| HAQ-DI (rheumatoid arthritis) | Cole et al., (2005) [196] | 0 | 0 | 0 | 0 | 0 | 0 | + | 0 | 0 | 0 | 0 |
| HAQ-DI (sclerderma) | Cole et al., (2006) [197] | 0 | 0 | 0 | 0 | 0 | 0 | + | 0 | 0 | 0 | 0 |
| HAQ-DI (rheumatoid arthritis) | Fries & Ramey (1997) [198] | 0 | 0 | 0 | 0 | 0 | 0 | 0 | + | - | 0 | 0 |
| HAQ-DI (rheumatoid arthritis) | Lawrence et al., (2009) [199] | 0 | 0 | 0 | 0 | 0 | 0 | 0 | ? | ? | 0 | 0 |
| HAQ-DI (rheumatoid arthritis) | Leigh & Fries (1992) [200] | 0 | 0 | 0 | 0 | 0 | 0 | 0 | + | 0 | 0 | 0 |
| HAQ-DI (rheumatoid arthritis) | Marra et al., (2005)[201] | 0 | 0 | 0 | + | 0 | 0 | 0 | 0 | + | 0 | 0 |
| HAQ-DI (rheumatoid arthritis) | Marra, Woolcott et al., (2005) [202] | 0 | 0 | 0 | 0 | 0 | 0 | 0 | + | 0 | 0 | 0 |
| HAQ-DI (systematic lupus erythematosus) | Milligan et al., (1993) [203] | + | 0 | 0 | 0 | 0 | 0 | + | + | 0 | 0 | 0 |
| HAQ-DI (scleroderma) | Poole et al., (1995) [204] | 0 | 0 | ? | 0 | 0 | 0 | ? | ? | 0 | 0 | 0 |
| HAQ-DI (rheumatoid arthritis) | Poole et al., (2006) [205] | 0 | 0 | 0 | 0 | 0 | 0 | 0 | + (AMPS also) | 0 | 0 | 0 |
| HAQ-DI (rheumatoid arthritis) | Ripat et al., (2001) [162] | 0 | 0 | 0 | 0 | 0 | 0 | 0 | + | 0 | 0 | 0 |
| HAQ-DI (rheumatoid arthritis) | Rohekar & Pope (2009) [206] | 0 | 0 | 0 | + | 0 | 0 | 0 | 0 | 0 | 0 | 0 |
| HAQ-DI (scleroderma) | Sultan et al., (2004) [207] | 0 | 0 | 0 | 0 | 0 | 0 | 0 | ? | 0 | 0 | 0 |
|  |  |  |  |  |  |  |  |  |  |  |  |  |
| Katz ADL (mixed) | Katz et al., (1963) [208] | 0 | 0 | ? | 0 | 0 | 0 | ? | ? | 0 | 0 | 0 |
| KATZ ADL (mixed) | Katz et al., (1970) [209] | 0 | 0 | 0 | 0 | 0 | ? | 0 | ? | 0 | 0 | 0 |
| KB ADL (spinal cord injury, stroke, traumatic brain injury) | Klein & Bell (1982) [210] | 0 | 0 | ? | 0 | 0 | 0 | 0 | ? | 0 | 0 | 0 |
| Lawton IADL (mixed) | Lawton & Brody (1969) [211] | 0 | 0 | + | 0 | 0 | 0 | 0 | + | 0 | 0 | 0 |
| Lifespace assessment mobility (community dwelling) | Baker et al., (2003) [212] | 0 | 0 | 0 | + | 0 | 0 | 0 | ? | 0 | 0 | 0 |
| Lifespace assessment mobility (community dwelling) | Crowe et al., (2008) [213] | 0 | 0 | 0 | 0 | 0 | 0 | 0 | + | 0 | 0 | 0 |
| Lifespace assessment mobility (community dwelling) | Peel et al., (2005) [214] | 0 | 0 | 0 | 0 | 0 | 0 | 0 | + | 0 | 0 | 0 |
| MBI (older adults) | de Morton et al., (2008) [102] | - | 0 | 0 | 0 | 0 | 0 | - | 0 | 0 | 0 | - |
| MBI (mixed) | Fricke & Unsworth (1996) [95] | 0 | 0 | 0 | ? | 0 | 0 | 0 | ? | 0 | 0 | 0 |
| MBI (stroke) | Hocking et al., (1999) [215] | 0 | 0 | 0 | 0 | 0 | 0 | 0 | 0 | ? | 0 | 0 |
| MBI (stroke) | Shah eet al., (1989) [111] | ? | 0 | 0 | 0 | 0 | 0 | 0 | 0 | + | 0 | 0 |
| MBI (traumatic brain injury) | Shah eet al., (2000) [216] | 0 | 0 | 0 | 0 | 0 | 0 | 0 | + | 0 | 0 | 0 |
| MBI (traumatic brain injury) | Shah & Muncer (2003) [217] | 0 | 0 | 0 | 0 | 0 | 0 | 0 | + | + | 0 | - |
| NEADL (stroke) | Ashburn et al., (2008) [218] |  |  |  |  |  |  |  |  |  |  |  |
| NEADL (stroke) | das Nair et al., (2011) [219] | 0 | 0 | 0 | 0 | 0 | 0 | + | 0 | 0 | 0 | 0 |
| NEADL (stroke) | Gladman et al., (1993) [108] | 0 | 0 | 0 | 0 | 0 | 0 | - | ? | 0 | 0 | 0 |
| NEADL (stroke) | Gompertz et al., (1993) [220] | 0 | 0 | 0 | + | + | 0 | 0 | 0 | 0 | 0 | 0 |
| NEADL (stroke) | Gompertz et al., (1994) [141] | 0 | 0 | 0 | 0 | 0 | 0 | 0 | ? | ? | 0 | 0 |
| NEADL (stroke) | Green et al., (2001) [140] | 0 | 0 | 0 | ? | - | 0 | 0 | 0 | 0 | 0 | 0 |
| NEADL (hip replacement) | Harwood & Ebrahim (2000) [104] | 0 | 0 | 0 | 0 | 0 | 0 | 0 | 0 | - | 0 | 0 |
| NEADL (hip replacement) | Harwood & Ebrahim (2002) [221] | + | 0 | 0 | + | 0 | 0 | - | 0 | - | 0 | 0 |
| NEADL (stroke) | Jacob-Lloyd et al., (2005) [222] | 0 | 0 | 0 | 0 | 0 | 0 | 0 | + | + | 0 | 0 |
| NEADL (stroke) | Lincoln & Gladman (1992) [223] | 0 | 0 | 0 | 0 | 0 | 0 | - | 0 | 0 | 0 | 0 |
| NEADL (stroke) | Nicholl et al., (2002) [224] | + | 0 | 0 | + | 0 | 0 | - | - | 0 | 0 | 0 |
| NEADL (stroke) | Nouri & Lincoln (1987) [225] | 0 | 0 | 0 | ? | 0 | 0 | - | 0 | 0 | 0 | 0 |
| NEADL (stroke) | Sarker et al., (2012) [149] | 0 | 0 | 0 | 0 | 0 | 0 | 0 | ? | 0 | 0 | 0 |
| RNLI (mixed) | Wood-Dauphinee et al., (1988) [226] | ? | 0 | + | 0 | 0 | + | + | + | + | 0 | 0 |
| Rivermead ADL (stroke) | Lincoln & Edmans (1990) [227] | 0 | 0 | 0 | 0 | 0 | 0 | + | 0 | 0 | 0 | 0 |
| Rivermead ADL (stroke) | Whiting & Lincoln., (1980) [228] | 0 | 0 | + | + | 0 | 0 | ? | 0 | 0 | 0 | 0 |
| SMAF (mixed) | Demers et al., (2010) [82] | 0 | 0 | 0 | 0 | 0 | 0 | 0 | 0 | + | 0 | 0 |
| SMAF (stroke) | Desrosiers et al., (2003) [85] | 0 | 0 | 0 | 0 | 0 | 0 | 0 | + | + | 0 | 0 |
| SMAF (older adults) | Hebert et al., (1997) [84] | 0 | 0 | 0 | 0 | + | 0 | 0 | 0 | 0 | 0 | 0 |
| SMAF (older adults) | Hebert et al., (1988) [83] | 0 | 0 | + | 0 | 0 | 0 | 0 | + | 0 | 0 | 0 |

* Studies shaded have a sample population of <50% of 70 years or older

AAP, Adelaide Activities Profile; ADL Index and IADL Index, Activities of Daily Living Index and Instrumental Activities of Daily Living Index; ADL Staircase, Activities of Daily Living Staircase; AMPS, Assessment of Motor and Process Skills; BI, Barthel Index; CAFU, Caregiver Assessment of Function and Upset; COPM, Canadian Occupational Performance Measure; DRI, Disability Rating Index; FAI, Frenchay Activity Index; FIM^TM^, Functional Independence Measure; FSQ Functional Status Questionnaire; GARS, Groningen Activity Restriction Scale; HAQ-DI Health Assessment Questionnaire –Disability Index; IAM, Instrumental Activity Measure; IDD, Interview of Deterioration in Daily Activities in Dementia; Katz ADL, Katz Activities of Daily Living; KB ADL, Klein Bell Activities of Daily Living; Lawton IADL, Lawton Instrumental Activities of Daily Living; MBI, Modified Barthel Index; Northwick Park Index of Independence in ADL, Northwick Park Index of Independence in Activities of Daily Living; NEADL, Nottingham Extended Activities of Daily Living; OBI, Original Barthel Index; RNLI, Reintegration to Normal Living Index; Rivermead ADL, Rivermead Activities of Daily Living assessment; SMAF, Functional Autonomy Measurement System; Sunnaas ADL, Sunnaas Activities of Daily Living Index.
