# Supplementary material for: Functional Assessments Used by Occupational Therapists with Older Adults at Risk of Activity and Participation Limitations: A Systematic Review
Source: PLoS One. 2016 Feb 9;11(2):e0147980. doi: 10.1371/journal.pone.0147980 (PMC4747506; doi:10.1371/journal.pone.0147980)
Supplement: S4 Information — (DOCX) [file pone.0147980.s004.docx]

**S4 Supporting Information. Data extracted on reliability.**

| **Assessment Tool** | **Reference** | **Result** |
| --- | --- | --- |
| AAP | Clark & Bond (1995) [106] | θ=0.80 domestic chores, 0.70 household maintenance, 0.52 service to others and 0.51 social activities. |
| BI | de Morton et al., (2008) [102] | Multidimensionality noted |
| BI (Collin and Wade) | Green et al., (2001) [140] | Mean difference 0.4 (95%CI 0.01-0.90) |
|  | Hartigan & O’Mahony (2011) [142] | Doctors and nurses, mean difference 1.2(95%CI =0.5-1.8 and LoA -5.483 to 7.975). Doctors and doctors mean difference 0.554 (-0.147 to 1.255 and LoA -5.1 to 6.2), nurses and nurses mean difference 0.154 (-0.397 to 0.705 and LoA -4.293 to 4.601). |
|  | Kidd eet al., (1995) [146] | Admission mean difference 0.8 (95%CI=-4.72 to 3.12), Discharge mean difference 0.44 (95%CI-2.02 to 2.9) |
| CAFU | Gitlin et al., (2005) [44] | Cronbachs α=0.83-0.91 |
| COPM | Jenkinson et al., (2007) [159] | r=0.53-0.67 for patients. |
|  | Sewell & Singh (2001) [164] | COPM performance r_s_=0.81 *p*<0.0001 and COPM satisfaction r_s_=0.76 *p*<0.001  COPM performance mean difference 0.14(95%CI-0.39 to 0.68) COPM satisfaction mean difference 0.42 (-0.18 to 1.01). |
| FAI | Carter et al., (1997) [92] | Mean difference 0.9 (95%CI of -5.4 to 7.5). |
|  | Green et al., (2001) [140] | Mean difference -0.6 (95%CI -2.21-0.93) |
|  | Turnbull et al., (2000) [170] | Relationship between time 1 and 2 r=0.96 |
| FIM | Dodds et al., (1993) [172] | Admission Cronbachs α=0.93 and discharge α=0.95 |
|  | Hamilton & Granger (1994) [175] | FIM ICC = 0.96 motor and 0.91 cognitive |
|  | Hobart et al., (2001) [96] | FIM ICC=0.98 |
|  | Jette et al., (2005) [97] | Cronbachs α=ADL 0.89; sphincter management=0.91; mobility = 0.76; executive function= 0.96) |
|  | Kidd et al., (1995) [146] | FIM admission mean 2.56 (95%CI-15.3-10.18), FIM discharge mean 0.64 (-16.8-18.08) |
|  | Kohler et al., (2010) [179] | FIM total scores ICC 0.872 (95%CI 0.822-0.908) |
|  | Ottenbacher et al., (1994) [181] | FIM total ICC = 0.92-0.99  FIM total ICC = 0.94-0.98 |
|  | Pollak et al., (1996) [182] | FIM motor ICC=0.90  FIM cognitive ICC=0.80 |
|  | Sharrack et al., (1999) [184] | Cronbachs α=0.98  Inter-rater FIM total ICC=0.99 intra-rater FIM total ICC=0.94, BI intra-rater ICC=0.98 |
|  | Stineman et al., (1996) [185] | Cronbach’s α =0.88-0.97 |
| FSQ | Reuben et al., (1995) [98] | Basic ADL cronbachs α=0.80 and Intermediate ADL α =0.81 |
| HAQ-DI | Marra, Rashidi et al., (2005) [201] | ICC=0.97 (95%CI 0.93-0.98) |
|  | Milligan et al., (1993) [203] | Cronbachs α=0.94 |
|  | Rohekar & pope (2009) [206] | ICC=0.897 (95%CI 0.855, 0.927) |
| Lawton IADL assessment | Lawton & Brody (1969) [211] | r =0.87-0.91 |
| Lifespace mobility | Baker et al., (2003) [212] | ICC=0.82-0.97. |
| MBI | de Morton et al., (2008) [102] | Multidimensionality noted, person separation index=0.91 |
| NEADL | Gompertz et al., (1994) [141] | Test retest correlations r_s_=0.92 *p*<0.05, Kappa ranged from fair to very good  Repeatability coefficient 2.8 out of 22. |
|  | Green et al., (2001) [140] | NEADL mean difference 0.6 (95%CI 0.75-1.85). Kappa 0.14-0.89, repeatability coefficient 5.6 out of 22  BI (Collin and Wade) mean difference 0.4(95%CI 0.01 to 0.90), Kappa 0-0.81, repeatability coefficient 2.0 out of 20  FAI mean difference -0.6(95%CI -2.21 to 0.93), Kappa 0.25-1.00, repeatability coefficient 7.1 (out of 45) |
|  | Harwood & Ebrahim (2002) [221] | Cronbachs α=0.90 (entire scale), ranged from 0.64-0.84 for subscales.  ICC=0.96 original scoring, ICC=0.97 likert scoring |
|  | Nicholl et al., (2002) [224] | Cronbachs α=0.94 (entire scale), ranged from 0.72-0.92 for subscales.  Test retest correlations r_s_=0.83-0.93 |
| RNLI | Woood-Dauphinee et al., (1988) [226] | Sample 1 patients and significant others r=0.621; patients and health professionals r=0.385 and health professionals and significant others r=0.340; Sample 2 patients and significant others r=0.648; patients and health professionals r=0.420; significant others and health professionals r=0.340 |
| Rivermead ADL assessment | Whiting & Lincoln (1980) [228] | Kendall coefficient of concordance =0.89  Correlation between time 1 and 2 r=0.95 |
| SMAF | Hebert et al., (1997) [84] | ±2.66 95%CI of ME 5.2. MDC is 5 point change on SMAF |
|  | Hebert eet al., (1988) [83] | Total weighted kappa 0.75 |

AAP, Adelaide Activities Profile; ADL, BI, Barthel Index; CAFU, Caregiver Assessment of Function and Upset; CI, Confidence Interval; COPM, Canadian Occupational Performance Measure; FAI, Frenchay Activity Index; FIM, Functional Independence Measure; FSQ, Functional Status Questionnaire; HAQ-DI, Health Assessment Questionnaire - Disability Index; ICC, Intraclass Correlation Coefficient; Lawton IADL, Lawton Instrumental Activities of Daily Living; LoA, Limits of Agreement; MBI, Modified Barthel Index, MDC, Minimally Detectable Change; ME, Measurement Error; NEADL, Nottingham Extended Activities of Daily Living; Rivermead ADL, Rivermead Activities of Daily Living assessment; RNLI, Reintegration to Normal Living Index; SMAF, Functional Autonomy Measurement System.
