# Supplementary material for: Functional Assessments Used by Occupational Therapists with Older Adults at Risk of Activity and Participation Limitations: A Systematic Review
Source: PLoS One. 2016 Feb 9;11(2):e0147980. doi: 10.1371/journal.pone.0147980 (PMC4747506; doi:10.1371/journal.pone.0147980)
Supplement: S5 Information — (DOCX) [file pone.0147980.s005.docx]

**S5 Supporting Information. Data extracted on validity.**

| **Tool** | **Reference** | **Result** |
| --- | --- | --- |
| AAP | Bond & Clark (1998) [105] | Discriminates between people using formal services, domestic chores |
|  | Clark& Bond (1995) [106] | Discriminates between patients on basis of domestic health and social circumstances  Supported four subscales. 45.3% of the total variance is explained. |
|  | Newson & Kemps (2005) [107] | AAP and cognitive assessments (picture naming, verbal fluency, incident recall and speed of processing) have a relationship (r=0.22-0.28*p*<0.001). |
| AMPS | Bernspang & Fisher (1995) [116] | Discriminates between people post stroke and healthy controls. |
|  | Doble et al., (1999) [89] | AMPS process and OARS *k*=0.36 |
|  | Duran & Fisher (1996) [117] | Overall the AMPS can be used across genders, although young women will perform better than men on AMPS process. |
|  | Fioravanti et al., (2012) [90] | AMPS motor and FIM motor r=0.54, AMPS process and FIM cognitive r=0.56 (*p*<0.001)  FIM motor and AMPS motor change scores (r=0.01 p=0.0951). FIM cognitive and AMPS process (r=0.19 p=0.0180). AMPS motor (*d*=2.14), AMPS process (*d*=1.59), FIM motor (*d=*2.47), FIM cognitive (*d=*0.57). AMPS process more sensitive than FIM cognitive (SRM change 0.74 *p*<0.05). |
|  | Hartman et al., (1999) [118] | Discriminates between people with dementia whom OT’s believe require community assistance. |
|  | Kottorp et al., (2003) [119] | Paces scoring criterion did not fit the model (Infit MnSq=1.5, z=9) for people with intellectual disability |
|  | Kottorp (2008) [120] | AMPS process has been shown to agree with clinical judgment of need for assistance in community for people with intellectual disability (OR 3.11, 95%CI 2.09-4.63). |
|  | McNulty & Fisher (2001) [121] | Delineate people who do not need assistance in the community (as assessed by clinical judgement) (80% predictive value, 78% sensitivity and 82% specificity).  Relationship between AMPS and SAFER r= 0.58-0.67 |
|  | Mercier et al., (2001) [86] | AMPS motor explained 64% of variance of functional autonomy in patients post-stroke. The SMAF explained 93% of variance, |
|  | Merritt & Fisher (2003) [122] | Free of gender bias |
|  | Merritt (2010) [123] | Maximum assistance, motor cut off 1.00 and process 0.70 |
|  | Merritt (2011) [124] | Motor cutoff 1.50 and 1.00 for process measures. Indicates needs for assistance in the community. |
|  | Pan & Fisher (1994) [125] | Discriminates between psychiatric and non psychiatric cases |
|  | Poole, Atanasoff, Poole, et al., (2006) [126] | Discriminates between people with and without systemic lupus erythematosus AMPS process t=-4.72 *p*<0.0001 and AMPS motor t=-4.83 *p*<0.0001. HAQ-DI also discriminates t=4.61 *p*<0.0001.. HAQ-DI not related to SLEDAI NSLEADI or AMPS tool.  Relationship between AMPS process and MMSE (r_s_=0.62), AMPS motor SLEDAI and neurological SLEDLAI (r_s_=-0.84 and r_s_=-0.77). |
|  | Robinson & Fisher (1996) [127] | Relationship between AMPS Process CAMCOG, MMSE (r=0.65 and r=0.67 *p*<0.001 respectively), AMPS Process and FIM cognitive (r=0.62 *p*<0.001), and AMPS motor and FIM motor (r=0.62 *p*<0.001) |
|  | Robinson & Fisher (1999) [128] | Can differentiate between people with and without dementia, AMPS motor t(27) 2.47 *p*=0.023; AMPS process skills t(27) 7.16 *p*<0.001 AMPS |
|  | White & Mulligan (2005) [129] | Can differentiate between children with and without Attention deficit hyperactivity disorder t(31)=3.47 *p*=0.001. |
|  | White et al., (2007) [131] | Can differentiate between the children with and without sensory processing difficulties, AMPS process (mean logit 0.08 SD=0.68) t(66)=-2.11 *p*=0.024 and AMPS motor mean logit 1.29 (0.52) t(66)=-2.08 *p*=0.025 |
|  | Stauffer et al., (2000) [129] | AMPS can be used across different American cultural groups |
| BI | Ahmed et al., (2003) [132] | BI scores at within 1 week post-stroke inform scores 3 months later  SRM at initial evaluation to 5 weeks 0.97 (95%CI 0.76-1.14) 5 weeks to 3month -0.42 (95%CI 0.07-0.62) initial to 3 month 0.9 (95%CI 0.60-1.10). |
|  | de Morton et al., (2008) [102] | Multidimensionality noted |
|  | Khan et al., (2008) [134] | BI and FIM change scores r_s_=0.74 *p*<0.001. BI ES=0.4 and SRM=0.8, FIM ES=0.4 and SRM=1.0 |
|  | Kwon et al., (2004) [135] | BI and m-FIM r_s_=0.9479 *p*<0.0001, BI and MRS r_s_=-0.8856 *p*<0.0001. |
|  | Wallace et al., (2002) [136] | All participants: paired t-test BI=12.1 and FIM 12, SRM BI=0.63 and FIM=0.62, ES BI=0.31 FIM=0.28, mixed model t-statistic BI=10.9, FIM=10.6. Changers: paired t-test BI=10.7 and FIM=11.6, SRM BI=0.86 and FIM=0.94; ES BI=0.48 and FIM=0.46; t-statistic BI=10.4 and 11.0 |
|  | Wellwood et al., (1995) [137] | OPCS and BI r=-0.73 *p*<0.001. With maximum and minimum scores eliminated correlation is r=-0.72 p<0.001. |
| BI (Collin and Wade) | Al-Khawaja et al., (1997) [138] | BI (C&W) and nursing hours r=-0.69 (95%CI -0.78 to -0.59). |
|  | Harwood & Ebrahim (2000) [104] | ES=0-0.08 |
|  | Hatfield et al., (2003) [143] | BI (C&W) and nursing time (part of NPDS) r_s_=-0.82 *p*<0.005, NPDS r_s_=-0.89 *p*<0.005, BI (C&W) and basic care needs (part of NPDS) r_s_=-0.95 and *p*<0.005. |
|  | Hobart et al., (2001) [96] | BI (C&W) and OPCS r=0.84, LHS r=0.33, SF-36PF r=0.30 ,SF-36MH r=0.11 ,GHQ r=0.14 and Wechsler Adult Intelligence r=0.28  BI (C&W), FIM and FIM+FAM r=0.96-0.996  SRM=0.56 |
|  | Hobart et al., (2010) [144] | Kazis ES BI (C&W) =-0.82 and FIM=-0.77; SRM BI (C&W)=-1.08 and FIM=-1.04 |
|  | Houlden et al., (2006) [145] | ES BI (C&W) =0.65 for all conditions, 0.79 for infarct, 0.52 intracerebral haemorrhage, 0.64 Subarachnoid haemorrhage and 0.55 for Traumatic brain injury. Correlation between BI (C&W) and FIM change scores r^2^=0.733 |
|  | Kidd et al., (1995) [146] | BI(C&W) and FIM *k=* 0.92 admission and *k=*0.88 at discharge.  BI(C&W) and FIM *k=*0.78 (change scores) |
|  | Van Der Putten et al., (1999) [150] | ES BI(C&W) =0.37 and FIM=0.30 in MS patients and ES BI (C&W) =0.95 and FIM=0.82 in stroke patients |
|  | Wade & Hewer (1987) [152] | BI (C&W) and morticity arm r=0.729, BI and leg r=0.751, BI (C&W) and total morticity index r=0.774  One major factor of the BI(C&W) |
|  | Wilkinson et al., (1997) [93] | BI (C&W) and LHS r_s_=0.726, FAI r_s_=0.826, SF-36 physical r_s_=0.810, social functioning r_s_=0.481, role physical r_s_=0.415, vitality r_s_=0.5, General health r_s_=0.438, NHP energy r_s_=-0.605, NHP pain r_s_=-0.499, NHP emotion r_s_=-0.423, and NHP social interaction r_s_=-0.460 , NHP physical mobility r_s_=-0.40, LSI total r_s_=0.361, SF-36 MH r_s_=0.332, Bodily pain r_s_=0.356 all *p*<0.001 ;LSI acceptance r_s_=0.307 *p*<0.01; sleep r_s_=-0.189 *p*>0.05 |
|  | Wright et al., (1998) [103] | BI (C&W) and RMI r_s_=0.74; 0.78; 0.87 *p*<0.001. RMI more responsive than BI (C&W)  ES=0.87 |
|  | Yohannes et al., (1998) [101] | BI (C&W) 19% sensitivity in identifying people with chronic airway limitation with a disability, NEADL 76% sensitivity |
| CAFU | Gitlin et al., (2005) [44] | Relationships with MMSE r_s_=-0.48 to -0.45 *p*<0.001, hours doing thing for care recipient r_s_=0.24-0.30 *p*<0.001, care giving r_s_=0.178 *p*<0.001, CES-D r=0.29-0.32 *p*<0.001, upset and problem behaviours r_s_=0.47 *p*<0.001 hours on duty r_s_=0.09-0.24 *p*≤0.05, problem behaviours r_s_=0.34*p*<0.001 and age was r_s_=0.14*p*<0.001  Two factor model |
| COPM | Bodiam (1999) [153] | COPM Performance and satisfaction increased between admission and discharge (r=0.69, *p*<0.01).  People with physical disability had more variation in scores (mean difference=2.70 performance, 4.50 satisfaction),  Satisfaction scores changed more than performance (t=8.60, *p*<0.001, satisfaction t=6.96, *p*<0.001) |
|  | Carpenter et al., (2001) [154] | COPM performance and satisfaction had a relationship with BAI, BDI, ODS, PSEQ, PVAS (at end of treatment and follow up, PVAS n/s for satisfaction at end of treatment).  COPM had significant differences between baseline, end of treatment and follow up *p*≤0.01. |
|  | Case-Smith (2003) [155] | COPM performance, DASH function and DASH symptoms (r=.656 *p*<0.001, r=0.557 *p*=0.001 respectively).  COPM satisfaction and DASH function and DASH symptoms (r=0.656 p<0.001, r=0.685 *p*<0.01 respectively).  COPM Performance (change) correlated with DASH function change and DASH symptoms (r=0.520 *p*=0.002 r=.500 *p*=0.003) COPM Satisfaction (change) correlated with DASH function change (r=.344 *p*=0.05)  ES COPM performance 2.45 COPM satisfaction 2.52 for satisfaction |
|  | Chan & Lee (1997) [156] | Assess occupational performance  No relationship between COPM, FIM and KB ADL assessment tools. COPM performance and SPSQ home management subscale (r=0.31 *p*<0.05) and COPM performance and SPSQ social community problem solving scores (r=0.39 *p*<0.05), COPM satisfaction , SPSQ social community problem solving scores (r=0.36*p*<0.05) and COPM performance and FIM motor r=0.32 *p*<0.05. |
|  | Donnelly et al., (2004) [157] | Admission FIM Motor and COPM performance r=0.452*p*<0.01 and COPM Satisfaction r=0.514 *p*<0.01; Discharge FIM motor and COPM performance r=0.388 p<0.05 and COPM Satisfaction r=0.513 *p*<0.05  COPM performance and FIM motor change scores (r=0.351*p*<0.05) and COPM satisfaction and FIM motor change scores (r=0.475 *p*<0.05) |
|  | Jenkinson eet al., (2007) [159] | COPM satisfaction and HADS anxiety (r=-0.42*p*<0.05)  COPM performance and satisfaction self ratings *p*=0.018 and 0.013 for patients, relatives assessment of performance was only significant p=0.008 |
|  | McColl et al., (2000) [160] | COPM satisfaction and SPSQ (r=0.394 *p*<0.05). COPM performance and RNLI (r=0.220 ns), COPM performance, COPM satisfaction and the LSS (r = 0.210 ns and r =0.463 *p*<0.01). SPSQ improved model fit (chi square=2.67, 1 df, *p*<0.5) and explained 21% of variance, RNLI was significant (t=2.23, df=1, *p*=0.03), and explained 13.4% of variance, LSS had significant effect t(t=2.57 p=0.01). |
|  | Law et al., (1994) [161] | COPM performance and satisfaction scores (r=0.76 *p*<0.001)  and change scores performance and satisfaction correlated (r=0.86 *p*<0.0001)  Mean change scores approximately 1.5 times improved. |
|  | Ripat et al., (2001) [162] | HAQ component and COPM performance r=-0.52*p*<0.01 and HAQ Activity and COPM performance r=-0.67*p*<0.01. |
|  | Rochman et al., (2008) [163] | COPM performance and PDI r=-0.62 to-0.75 *p*≤0.05 and GVAS r=-0.39 to r=-0.42 *p*≤0.05  COPM satisfaction and PDI r=-0.41 to-0.79 *p*≤0.05 and GVAS r=-0.28 ns to -0.93 *p*<0.05  COPM satisfaction had significant change *t=*5.84p<0.0001. not significant for performance |
|  | Stuber & Nelson (2010) [165] | COPM performance and OSA (r=0.51 *p*=0.0038), SIGA (r=0.58, *p*=0.0008), SIGA (mean of identified goals) r=0.76 *p*<0.0001, COPM satisfaction OSA (r=0.54 *p*=0.0021) and SIGA overall (r=0.62 *p*=0.0003) |
|  | Walsh et al., (2004) [167] | COPM performance and RMDQ r_s_=-0.25ns to -0.31*p*<0.01 and self efficacy r_s_=0.25 to 0.46 *p*≤0.05. COPM satisfaction and RMDQ ns and self efficacy r_s_=0.20 to0.30 *p*<0.05.  COPM changed throughout program *p*<0.0001 |
|  | van Huet & Williams (2007) [166] | COPM discriminated between those who did and did not improve (COPM performance t=4.434, df=62, p<0.05, COPM satisfaction t=4.02, df=62, *p*<0.05). |
|  |  |  |
| FAI | Carter et al., (1997) [92] | Postal and interview assessment r_s_=0.94 *p*<0.0001 |
|  | Holbrook & Skilbeck (1983) [168] | Three major factors explain 52% of the variance supporting the structure of the tool. |
|  | Patel et al., (2006) [169] | BI (C&W) and FAI at 1 year r_s_=0.83 and 3 years r_s_=0.79 |
|  | Wilkinson et al., (1997) [93] | FAI & BI(C&W) r_s_=0.80 (0.74-0.84), BI and NEADL r_s_=0.88 (0.84-0.90) NEADL and FAI r_s_=0.90 (0.88-0.92). |
| FIM | Corrigan et al., (1997) [171] | FIM predictive of minutes of assistance (*p*<0.0001), supervision (*p*<0.0063) and need other type of assistance (*p*<0.0032). |
|  | Dodds et al., (1993) [172] | Admission and discharge FIM scores decreased with increasing age, co-morbidities. FIM scores discriminated across different impairment severities (*p*<0.005).  Improvements between admission and discharge FIM scores (*p*<0.005). |
|  | Hall et al., (1993) [94] | FIM Motor correlated with Disability rating scale (DRS r=0.641), FIM and FAM motor (r=0.992) and FIM and FAM cognition (r+0.653); FIM cognition with DRS (r=0.728) FIM and FAM motor (r=0.645) and FIM and FAM cognition (r=0.635). |
|  | Graves (2005) [174] | Factor analysis of FIM data produced a two factor oblique solution accounting for 81% of the variation. Correlation between 2 FIM factors in solution is 0.642 (moderate to high relation). |
|  | Heinemann et al., (1993) [176] | Supported FIM as an interval measure and supported a motor and cognitive subscale. Differences between subgroups as expected. |
|  | Heinemann et al., (1994) [177] | 97.9% of the variance was accounted for. |
|  | Hobart et al., (2001) [96] | BI (C&W) and OPCS r=0.82, LHS r=0.32, SF-36PF r=0.26 ,SF-36MH r=0.10 ,GHQ r=0.13 and Wechsler Adult Intelligence r=0.35  BI (C&W), FIM and FIM+FAM r=0.96-0.996, SRM=0.48 |
|  | Houlden et al., (2006) [145] | FIM total ES (0.52-0.0.72). ES FIM cognitive score (0.35-0.43) |
|  | Jette et al., (2005) [97] | Corrected item-total correlations 0.58-0.80 for ADL domain, from 0.23-0.71 mobility domain, from 0.78-0.88 executive function domain. 73.4% of variance explained. Correlation for sphincter management was 0.84. Transferring into shower/tub was related to ADL domain rather than mobility. |
|  | Kidd et al., (1995) [146] | BI(C&W) and FIM *k=* 0.92 admission and *k=*0.88 at discharge.  BI(C&W) and FIM *k=*0.78 (change scores) |
|  | Linacre et al., (1994) [180] | FIM total score – not supported, motor and cognitive subscale supported. Cognitive has no misfitting items, motor misfitting items (eating, bladder & bowel management, stairs) Further revision to the FIM motor score required. |
|  | Pollak et al., (1996) [182] | Significant differences between people in skilled nursing facility, sheltered care and independent community FIM motor scores *F*(2,46)=34.71 *p<*0.05 and FIM cognitive *F*(2,46)=12.42 *p*<0.05. (note toileting was omitted)  Bladder and bowel management and grooming misft and cognitive memory. Supported motor and cognitive subscales. |
|  | Sharrack et al., (1999) [184] | Supported 2 factor structure, 89.4% of total variance explained.  FIM correlated with BI(C&W) r=0.88, EDSS r=0.74 SNRS r=0.69 AI r=-0.72 and CAMBS r=-0.69 to -0.61 respectively and SF-36 (*r*=0.88) (all *p*<0.001).FIM correlated with work r=-0.59 (*p<*0.001), housework r=-0.64 (*p*=0.001), independence (r=-0.44 *p*=0.001) and disability rank r=-0.96 *p<*0.001  FIM total score (ES= 0.46, *p<*0.001). Cognitive items not responsive. BI (C&W) (ES=0.25 *p*=0.042) |
|  | Stineman et al., (1996) [185] | Item -total correlations 0.26-0.91 (FIM motor) and 0.60-0.87 (FIM cognitive). Supported the motor and cognitive subscales but also raised that a 3 or 4 factor structure was possible.>50% of variance explained in following subgroups; stroke, non traumatic brain injury, traumatic brain injury, non traumatic spinal cord injury, traumatic spinal cord injury, Gullian Barre, general neurological, lower extremity fracture, joint replacement, other orthopaedic, lower extremity amputation, other amputation, osteoarthritis, rheumatoid arthritis, cardiac, pulmonary, pain, major multiple trauma, multiple trauma with brain and spine, others. |
| FSQ | Jette et al., (198) [186] | Basic ADL and Intermediate ADL and age, bed days, restricted activity days, satisfaction with health, work role limitations, number of close friends/frequency of social contact (r=-0.28, -0.33, 0.35, -0.44, 0.07 AND -0.15, -0.26, -0.36, 0.43, -0.61, 0.007 *p<*0.001). |
|  | Katz et al., (1992) [187] | Global FSQ and SIP r=0.82, SF-36 r=0.83, sAIMS r=0.76. Physical dimensions FSQ and SIP r=0.76, SF-36 r=0.73, MHAQ r=0.76 sAIMS 0.68.  Basic ADL SRM =1.10, intermediate ADL=0.89 |
|  | Reuben et al., (1992) [188] | Falling into the intermediate ADL and social activities warning zones was significantly associated with death. IADL r=-0.218 *p<*0.01; social activities r=-0.25 *p<*0.01. |
|  | Reuben et al., (1995) [98] | FSQ basic ADL and SF-36 PF-10 r=0.51; FSQ IADL and SF-36 PF10 r=0.76 . FSQ BADL and modified Katz r=0.47 and OARS r=0.70. FSQ intermediate ADL and modified katz r=0.31 and OARS r=0.59. FSQ BADL and PPT r=0.55 and FSQ Intermediate ADL and PPT r=0.45 . FSQ Basic ADL to SF-36 to subscales r=0.33 (Emotional wellbeing) to r=0.53 (general health). FSQ intermediate ADL and SF-36 subscales r=0.35(emotional) to 0.59 (energy/fatigue). All *p<*0.001 |
|  | Rubenstein et al.,(1998) [189] | FSQ basic ADL and SF-36 r=0.30-0.75, FSQ intermediate ADL r=0.31-0.77 (all *p<*0.001), FSQ basic ADL and UPDRS r=0.70, FSQ intermediate ADL r=0.73 (all p≤0.0545). |
|  | Yarnold et al., (1995) [190] | Comparable variability between non geriatrics (*χ*^2^ df=15, n=115) =14.0 *p<*0.53) and geriatric population (*χ*^2^ df=15, n=106) =31.7*p<*0.008) |
| HAQ-DI | Benton et al., (2004) [191] | Baseline HAQ-DI correlated with MR bone odema (r_s_=0.44 p=0.004). At 6yrs HAQ-DI and total MR score correlated (r_s_=0.48 p=0.0065), and bone erosion (r_s_=0.48 p=0.007), and SF-36 PF (r_s_=-0.725 *p<*0.0001). |
|  | Bombardier & Raboud (1991) [192] | ES = 0.25. |
|  | Bruce & Fries (2004) [193] | HAQ-DI correlated with WOMAC function rs=0.71 to 0.79, WOMAC stiffness rs=0.61 to 0.64, patient global assessment rs=0.59 to 0.65.  HAQ-DI ES= 0.2. HAQ-DI change scores correlated with WOMAC function, pain and stiffness (rs=0.44, rs=0.28 rs=0.30 respectively), global health rs=0.345 (all significant at *p<*0.0001) |
|  | Buchbinder et al., (1995) [194] | Correlation of change scores, HAQ-DI and tender joint count had an overall correlation of r=0.41 and a correlation of r=0.54 for active treatment and r=0.21 for placebo. |
|  | Clements et al., (2001) [195] | HAQ-DI score of ≥1.0 was predictive of mortality (OR 3.22 95%CI 1.097-9.468) |
|  | Cole et al., (2005) [196] | Obtained a single factor structure for HAQ-DI |
|  | Cole et al., (2006) [197] | Obtained a two factor structure HAQ-DI but single factor ok |
|  | Fries & Ramey (1997) [198] | HAQ-DI and pain scale correlated r=0.50 *p<*0.001 Global Analogue scale r=0.56 *p<*0.001 and Feeling thermometer r=-0.51*p<*0.001  HAQ-DI change scores and pain r=0.37*p<*0.013 and Global Analogue scale r=0.36 *p<*0.017 and Feeling thermometer r=-0.33 *p<*0.032. |
|  | Leigh & Fries (1992) [200] | HAQ-DI is predictive of HAQ-DI (correlation coefficient 0.77*p<*0.001) (8 years later). |
|  | Marra, Rashidi et al., (2005) [201] | Worse HAQ-DI score ES =0.22 (95%CI 0.04-0.38); Same=-0.9(95%CI-0.28-0.02) Better=-0.24 (95%CI -0.38 to -0.11). SRM also reported. Worse SRM= 0.33 (95%CI 0.06-0.65); Same=-0.20 (95%CI -0.56 to -0.10) and better=-0.39 (95%CI -0.69 to -0.30). |
|  | Marra, Woolcott, et al., (2005) [202] | Correlation between HAQ-DI and self reported RA r_s_=0.46, self reported RA control r_s_=0.45 (all *p<*0.001). MID=0.25 |
|  | Milligan et al., (1993) [203] | HAQ-DI correlated and ARA criteria r=-0.23, lupus activity criteria count r=0.22, number of reported symptoms r=0.53, number of doctor visits in 6 months r=0.29, patient rating of health r=0.59 (all *p<*0.001), non significant disease severity.  2 factor structure |
|  | Poole, Atanasoff, et al., (2006) [126] | HAQ-DI and DHI had a strong correlation (r_s_=0.78 *p*=0.01) |
|  | Ripat eet al., (2001) [162] | HAQ-DI component and COPM performance r=-0.52*p<*0.01 and HAQ Activity and COPM performance r=-0.67*p<*0.01. |
| KB ADL Scale | Klein & Bell (1982) [210] | A lower score at baseline indicated a greater amount of assistance in the community. |
| Lawton IADL | Lawton & Brody (1969) [211] | r=0.61 PSMS, r=0.40 PC, r=0.48 MSQ *p<*0.01 |
| Lifespace assessment (mobility) | Crowe et al., (2008) [213] | Assessment is indicative of cognition four years later. |
|  | Peel et al., (2005) [214] | Significant correlations with MMSE (r=0.40), GDS score (r=-0.39), ADL limitations (r=-0.20 to -0.49), IADL limitations (r=-0.16 to -0.55) and SPPB (r=-0.51 to 0.63) (all *p<*0.0001). |
| MBI | de Morton et al., (2008) [102] | Not a unidimensional tool |
|  | Shah et al., (1989) [111] | Modifications to 75 improved sensitivity. MBI Mean correlation= 0.5385-0.6665, min correlation= 0.1476-0.2830, max correlation=0.8361-0.9012 BI Mean correlation= 0.4287-0.5697, minimum correlation= 0.1221-0.2584, maximum correlation=0.7781-0.8375 |
|  | Shah et al., (2000) [216] | Stepwise regression: delay in commencing rehabilitation and age contributed to 68% of variance |
|  | Shah & Muncer (2003) [217] | MBI Predictive of length of stay in rehabilitation and hospital.  MBI and Disability rating scale (r=-0.765 *p*<0.001)  Change score, t=7.60 p=0.001, ES=0.63 |
| NEADL | Ashburn et al., (2008) [218] | NEADL score is not predictive of falls post-stroke. |
|  | das Nair et al., (2011) [219] | NEADL is not uni-dimensional. Rescoring of subscales and removal of 4 items improved fit. Questions “manage to feed self” and “manage money when out” “walk around outside” and “wash small items of clothing” were removed. Kitchen and domestic subscales combined. |
|  | Gladman et al., (1993) [108] | Did not form adequate guttman scale until following question was dropped “do you manage your own money when you are out” |
|  | Harwood & Ebrahim (2000) [104] | At 3months ES=0.02-0.22 and 12months=0.05-0.34 |
|  | Harwood & Ebrahim (2002) [221] | Did not form hierarchical Guttmann model  ES were poor at 3 and 6 months. 3months: total ES=0.14, mobility ES=0.22, domestic ES=0.04, kitchen ES=0.11, leisure ES=0.17 6 months: total ES=0.27, mobility ES=0.30, domestic ES=0.05, kitchen ES=0.09, leisure ES=0.34 |
|  | Jacob-Lloyd et al., (2005) [222] | MBI and NEADL baseline r_s_=0.71; r_s_=0.88 *p<*0.01  ES BI=0.17 and NEADL=0.63 |
|  | Lincoln & Gladman (1992) [223] | Kitchen and domestic tasks could form one subscale. |
|  | Nicholl et al., (2002) [224] | NEADL correlated with GNDS total r_s_=-0.57, SF-36PF r_s_=0.25, SF-36MH r_s_=0.26, OQoL r_s_=0.36 and SQoL r_s_=0.36 (all *p<*0.001)  Not a uni-dimensional scale. |
|  | Nouri & Lincoln (1987) [225] | *K=*0.29-1.00  Uni-dimensional (except for mobility subscale) |
| RNLI | Wood-Dauphinee et al., (1988) [226] | Agreement that RNLI is valid  RNLI correlated with QL index r=0.721 and at follow up r=0.549 (all *p<*0.05).  Two factors, daily functioning and perceptions of self  r=0.560*p<*0.05 (patients); r=0.359 *p<*0.05 (sig others) |
| Rivermead ADL | Lincoln & Edmans (1990) [227] | Coefficient of reproducibility = 0.89-0.99 and coefficient of scalability = 0.89-0.98 |
| SMAF | Demers et al., (2010) [82] | SMAF ADL Geriatric rehabilitation Unit and Day hospital (SRM and Guyatt Effect sizes (GRU 0.97-2.17; DH 0.29-0.54); externally responsive to health professionals perception of change in participants (partial n^2^ 0.19-0.20 *p*<0.05) |
|  | Desrosiers et al., (2003) [85] | SMAF correlated with FIM total score during and after rehabilitation SMAF and FIM r=0.93-0.95 (*p*<0.001). SMAF is more correlated to the LIFE-H than the FIM SMAF and LIFE-H r=0.85-0.89 and FIM and LIFE H r=0.79-0.85. Both SMAF and FIM will have low to moderate correlations with LIFE-H social roles. SMAF and LIFE-H r=0.66-0.77 FIM and LIFE-H r=0.57-0.71  SMAF (without IADL component) SRM 1.04 (95%CI 0.82, 1.26); SMAF with IADL component SRM 1.20 (0.98, 1.42), FIM SRM 0.97 (0.76, 1.28) |
|  | Hebert et al., (1997) [84] | SMAF and nursing care time r=0.58-0.89*p<*0.0001 |

AAP, Adelaide Activities Profile; ADL, Activities of Daily Living; AMPS, Assessment of Motor and Process Skills; ARA, American Rheumatism Criteria; BAI, Beck Anxiety Inventory; BDI, Beck Depression Inventory; BI, Barthel Index; CAFU, Caregiver Assessment of Function and Upset; CAMBS, Cambridge Multiple Sclerosis Basic Score; CAMCOG, Cambridge Cognition Examination; CESD, Centre for Epidemiological Studies Depression; CI, Confidence Interval; COPM, Canadian Occupational Performance Measure; DASH, Disability of the Arm Shoulder and Hand; DHI, Duruox Hand Index; DRS, Disability Rating Scale; EDSS, Expanded Disability Status Scale; ES, Effect size; FAI, Frenchay Activity Index; FAM, Functional Assessment Measure; FIM, Functional Independence Measure; FSQ, Functional Status Questionnaire; GHQ, General Health Questionnaire; GNDS, Guy's Neurological Disability Scale; GVAS, Global Visual Analogue Scale; HADS, Hospital Anxiety Depression Scale; HAQ-DI, Health Assessment Questionnaire - Disability Index; IADL, Instrumental Activities of Daily Living; KB ADL Scale, Klein Bell ADL Scale; LHS, London Handicap Scale; Life-H, The Assessment of Life Habits; LSI, Life Satisfaction Index; MBI, Modified Barthel Index; MHAQ, Modified Health Assessment Questionnaire; MMSE, Mini Mental State Examination; MnSq, Mean Square; MSQ, Mental Status Questionnaire; NEADL, Nottingham Extended Activities of Daily Living; NHP, Nottingham Health Profile; NPDS, Northwick Park Dependency Score; NS, non-significance; NSLEADI, Non Systemic Lupus Erythematosus Disease; OARS, Older Americans Resources and Services ODS, Oswestry Disability Index; OPCS, Office of Population Censuses and Surveys; OQoL, Overall Quality of Life; OR, Odds Ratio; OSA, Occupational Self Assessment; OT's, Occupational Therapists; PC, Physical Classification; PDI, Pain Disability Index; PSEQ, Pain Self-Efficacy Questionnaire; PSMS, Physical Self Maintenance Scale; PVAS, Pain Visual Analogue Scale; QL, Quality of Life; RA, Rheumatoid Arthritis; Rivermead ADL, Rivermead Activities of Daily Living; RMDQ, Roland-Morris Disability Questionnaire; RMI, Rivermead Motor Index; RNLI, Reintegration to Normal Living Index; SAFER, Safety Assessment of Function and the Environment for Rehabilitation; sAIMS, Shortened Arthritis Impact Measure; SD, Standard Deviation; SF-36, Short Form Health Survey; SIGA, Melville-Nelson Self-Identified Goals Assessment; SLEDAI, Systemic Lupus Erythematosus Disease Activity Index; SMAF, Functional Autonomy Measurement System; SNRS, Scripps Neurological Rating Scale; SPSQ, Satisfaction with Performance Scaled Questionnaire; SQoL, Subjective Quality of Life; SRM, Standardised response mean; UPDRS, Unified Parkinson's Disease Rating Scale; WOMAC, Western Ontario and McMaster Universities Arthritis Index
