# Supplementary material for: Functional Assessments Used by Occupational Therapists with Older Adults at Risk of Activity and Participation Limitations: A Systematic Review
Source: PLoS One. 2016 Feb 9;11(2):e0147980. doi: 10.1371/journal.pone.0147980 (PMC4747506; doi:10.1371/journal.pone.0147980)
Supplement: S6 Information — (DOCX) [file pone.0147980.s006.docx]

**S6 Supporting Information. Complete reference list.**

1. Australian Bureau of Statistics. 2071.0 - Reflecting a Nation: Stories from the 2011 Census, 2012–2013 2012 [cited 2014 May 20th]. Available from: http://www.abs.gov.au/ausstats/abs@.nsf/Lookup/2071.0main+features752012-2013.

2. Office for National Statistics. What does the 2011 Census tell us about people? 2013 [cited 2014 10th May]. Available from: http://www.ons.gov.uk/ons/rel/census/2011-census-analysis/what-does-the-2011-census-tell-us-about-older-people-/what-does-the-2011-census-tell-us-about-older-people--full-infographic.html.

3. U.S. Department of Health and Human Services. A Profile of older Americans 2013 2013 [cited 2014 May 10]. Available from: http://www.aoa.gov/Aging_Statistics/Profile/Index.aspx.

4. Fawcett AJL. Principles of assessment and outcome measures for occupational therapists and physiotherapists: Theory, skills and application. Chichester: John Wiley & Sons; 2007.

5. Unsworth C. Measuring the outcome of occupational therapy: Tools and resources. Australian Occupational Therapy Journal. 2000;47(4):147-58. doi: 10.1046/j.1440-1630.2000.00239.x.

6. Bowman J. Challenges to measuring outcomes in occupational therapy: A qualitative focus group study. British Journal of Occupational Therapy. 2006;72:55-64.

7. Burton L-J, Tyson S, McGovern A. Staff perceptions of using outcome measures in stroke rehabilitation. Disability and Rehabilitation. 2012;35(10):828-34. doi: 10.3109/09638288.2012.709305.

8. Fricke J, Unsworth CA. Occupational Therapists' Conceptions of Instrumental Activities of Daily Living in Relation to Evaluation and Intervention with Older Clients. Scandinavian Journal of Occupational Therapy. 1998;5(4):180-91. doi: doi:10.3109/11038129809035744.

9. Law M, Letts L. A critical review of activties of daily living. American Journal of Occupational Therapy. 1989;43:522-8.

10. Mayo N, Cole B, Dowler J, Gowland C, Finch E. Use of outcome measures in physiotherapy: A survey of current practice. Canadian Journal of Rehabilitation. 1993;7:81-2.

11. Van Peppen RPS, Maissan FJF, Van Genderen FR, Van Dolder R, Van Meeteren NLU. Outcome measures in physiotherapy management of patients with stroke: a survey into self-reported use, and barriers to and facilitators for use. Physiotherapy Research International. 2008;13(4):255-70. doi: 10.1002/pri.417.

12. Buurman BM, van Munster BC, Korevaar JC, de Haan RJ, de Rooij SE. Variability in measuring (instrumental) activities of daily living functioning and functional decline in hospitalized older medical patients: a systematic review. Journal of clinical epidemiology. 2011;64(6):619-27.

13. Latham Trombly C. A. Conceptual foundations for practice. Occupational therapy for physical dysfunction. 6th ed. Baltimore: Lippincott Williams and Wilkins; 2008.

14. Carswell A, Donnelly C. Individualised outcome measures: A review of literature. Canadian Journal of Occupational Therapy. 2002;69:84-100.

15. Australian Government. 2015 Intergenerational Report: Australia 2015 Canberra: Commonwealth of Australia; 2015 [cited 2015 4th November ]. Available from: http://www.treasury.gov.au/PublicationsAndMedia/Publications/2015/2015-Intergenerational-Report.

16. World Health Organization. Towards a common language for functioning, disability and health (ICF) Geneva2002 [cited 2014 20th May].

17. Hemmingsson H, Jonsson H. An occupational perspective on the concept of participation in the international claassification of functioning, disability and health - some critical remarks. American Journal of Occupational Therapy. 2005;59:569-76.

18. Stamm TA, Cieza A, Machold K, Smolen JS, Stucki G. Exploration of the link between conceptual occupational therapy models and the International Classification of Functioning, Disability and Health. Australian Occupational Therapy Journal. 2006;53(1):9-17. doi: 10.1111/j.1440-1630.2005.00513.x.

19. Wales K, Clemson L, Lannin N, Cameron I. Functional assessments used by occupational therapists with older adults at risk of activity and participation limitations: a systematic review and evaluation of measurement properties. Systematic Reviews. 2012;1(1):45. PubMed PMID: doi:10.1186/2046-4053-1-45.

20. Mokkink LB, Terwee CB, Patrick DL, Alonso J, Stratford PW, Knol DL, et al. Cosmin checklist manual2012 01-02-13:[1-56 pp.]. Available from: http://www.cosmin.nl/the-cosmin-checklist_8_5.html.

21. Mokkink LB, Terwee CB, Patrick DL, Alonso J, Stratford PW, Knol DL, et al. The COSMIN study reached international consensus on taxonomy, terminology, and definitions of measurement properties for health-related patient-reported outcomes. Journal of Clinical Epidemiology. 2010;63(7):737-45. doi: http://dx.doi.org/10.1016/j.jclinepi.2010.02.006.

22. Dobson F, Hinman RS, Hall M, Terwee CB, Roos EM, Bennell KL. Measurement properties of performance-based measures to assess physical function in hip and knee osteoarthritis: a systematic review. Osteoarthritis and Cartilage. 2012;20(12):1548-62. doi: http://dx.doi.org/10.1016/j.joca.2012.08.015.

23. Terwee CB, Bot SDM, de Boer MR, van der Windt DAWM, Knol DL, Dekker J, et al. Quality criteria were proposed for measurement properties of health status questionnaires. Journal of Clinical Epidemiology. 2007;60(1):34-42. doi: http://dx.doi.org/10.1016/j.jclinepi.2006.03.012.

24. Brédart A, Kop JL, Efficace F, Beaudeau A, Brito T, Dolbeault S, et al. Quality of care in the oncology outpatient setting from patients' perspective: a systematic review of questionnaires' content and psychometric performance. Psycho-Oncology. 2015;24(4):382-94. doi: 10.1002/pon.3661.

25. Zwakhalen SM, Hamers JP, Abu-Saad HH, Berger MP. Pain in elderly people with severe dementia: A systematic review of behavioural pain assessment tools. BMC Geriatrics. 2006;6(3):1-15.

26. Anderson C, Rubenach S, Mhurchu CN, Clark M, Spencer C, Winsor A. Home or hospital for stroke rehabilitation? results of a randomized controlled trial : I: health outcomes at 6 months. Stroke. 2000;31(5):1024-31. PubMed PMID: 10797161.

27. Gitlin LN, Winter L, Dennis MP, Corcoran M, Schinfeld S, Hauck WW. A randomized trial of a multicomponent home intervention to reduce functional difficulties in older adults. Journal of the American Geriatrics Society. 2006;54(5):809-16. PubMed PMID: 16696748.

28. Gitlin LN, Winter L, Dennis MP, Hauck WW. Variation in response to a home intervention to support daily function by age, race, sex, and education. Journals of Gerontology Series A-Biological Sciences & Medical Sciences. 2008;63(7):745-50. PubMed PMID: 18693230; PubMed Central PMCID: PMCNIHMS99124

PMC2656911.

29. Berggren M, Stenvall M, Olofsson B, Gustafson Y. Evaluation of a fall-prevention program in older people after femoral neck fracture: a one-year follow-up. Osteoporosis International. 2008;19(6):801-9. PubMed PMID: 18030411.

30. Eklund K, Sjostrand J, Dahlin-Ivanoff S. A randomized controlled trial of a health-promotion programme and its effect on ADL dependence and self-reported health problems for the elderly visually impaired. Scandinavian Journal of Occupational Therapy. 2008;15(2):68-74. PubMed PMID: 2009972172. Language: English. Entry Date: 20081003. Revision Date: 20101231. Publication Type: journal article.

31. Crotty M, Giles LC, Halbert J, Harding J, Miller M. Home versus day rehabilitation: a randomised controlled trial. Age & Ageing. 2008;37(6):628-33. PubMed PMID: 18723862; PubMed Central PMCID: PMCPMC2582455.

32. Graff MJ, Vernooij-Dassen M, Thijssen M, Dekker J, Hoefnagels WH, Rikkert MG. Community based occupational therapy for patients with dementia and their care givers: randomised controlled trial. British Medical Journal. 2006;333(7580):1196.

33. Conroy S, Kendrick D, Harwood R, Gladman J, Coupland C, Sach T, et al. A multicentre randomised controlled trial of day hospital-based falls prevention programme for a screened population of community-dwelling older people at high risk of falls. Age & Ageing. 2010;39(6):704-10. doi: 10.1093/ageing/afq096. PubMed PMID: 2010837083. Language: English. Entry Date: 20101105. Revision Date: 20120120. Publication Type: journal article.

34. Corr S, Bayer A. Occupational therapy for stroke patients after hospital discharge -- a randomized controlled trial. Clinical Rehabilitation. 1995;9(4):291-6. PubMed PMID: 1996007072. Language: English. Entry Date: 19960301. Revision Date: 20091218. Publication Type: journal article.

35. Cunliffe AL, Gladman JR, Husbands SL, Miller P, Dewey ME, Harwood RH. Sooner and healthier: a randomised controlled trial and interview study of an early discharge rehabilitation service for older people. Age and Ageing. 2004;33(3):246-52.

36. Fleming SA, Blake H, Gladman JRF, Hart E, Lymbery M, Dewey ME, et al. A randomised controlled trial of a care home rehabilitation service to reduce long-term institutionalisation for elderly people. Age & Ageing. 2004;33(4):384-90. PubMed PMID: 15151912.

37. Lincoln NB, Walker MF, Dixon A, Knights P. Evaluation of a multiprofessional community stroke team: a randomized controlled trial. Clinical Rehabilitation. 2004;18(1):40-7. PubMed PMID: 14763718.

38. Logan PA, Gladman JRF, Avery A, Walker MF, Dyas J, Groom L. Randomised controlled trial of an occupational therapy intervention to increase outdoor mobility after stroke.[Erratum appears in BMJ. 2005 Jan 15;330(7483):137]. BMJ. 2004;329(7479):1372-5. PubMed PMID: 15564229; PubMed Central PMCID: PMCPMC535450.

39. Parker CJ, Gladman JRF, Drummond AER, Dewey ME, Lincoln NB, Barer D, et al. A multicentre randomized controlled trial of leisure therapy and conventional occupational therapy after stroke. Clinical Rehabilitation. 2001;15(1):42-52. PubMed PMID: 2001063953. Language: English. Entry Date: 20010720. Revision Date: 20091218. Publication Type: journal article.

40. Richards SH, Coast J, Gunnell DJ, Peters TJ, Pounsford J, Darlow MA. Randomised controlled trial comparing effectiveness and acceptability of an early discharge, hospital at home scheme with acute hospital care.[Erratum appears in BMJ 1998 Sep;317(7161):786]. BMJ. 1998;316(7147):1796-801. PubMed PMID: 9624070; PubMed Central PMCID: PMCPMC28580.

41. Roderick P, Low J, Day R, Peasgood T, Mullee MA, Turnbull JC, et al. Stroke rehabilitation after hospital discharge: a randomized trial comparing domiciliary and day-hospital care. Age & Ageing. 2001;30(4):303-10. PubMed PMID: 11509308.

42. Rudd AG, Wolfe CD, Tilling K, Beech R. Randomised controlled trial to evaluate early discharge scheme for patients with stroke.[Erratum appears in BMJ 1998 Feb 7;316(7129):435]. BMJ. 1997;315(7115):1039-44. PubMed PMID: 9366727; PubMed Central PMCID: PMCPMC2127677.

43. Gitlin LN, Corcoran M, Winter L, Boyce A, Hauck WW. A randomized, controlled trial of a home environmental intervention: effect on efficacy and upset in caregivers and on daily function of persons with dementia. Gerontologist. 2001;41(1):4-14. PubMed PMID: 2001054405. Language: English. Entry Date: 20010615. Revision Date: 20091218. Publication Type: journal article.

44. Gitlin LN, Roth DL, Burgio LD, Loewenstein DA, Winter L, Nichols L, et al. Caregiver appraisals of functional dependence in individuals with dementia and associated caregiver upset: psychometric properties of a new scale and response patterns by caregiver and care recipient characteristics. Journal of Aging & Health. 2005;17(2):148-71. PubMed PMID: 2005098676. Language: English. Entry Date: 20050624. Revision Date: 20091218. Publication Type: journal article.

45. Clare L, Linden DEJ, Woods RT, Whitaker R, Evans SJ, Parkinson CH, et al. Goal-oriented cognitive rehabilitation for people with early-stage alzheimer disease: A single-blind randomized controlled trial of clinical efficacy. American Journal of Geriatric Psychiatry. 2010;18(10):928-39.

46. Hagsten B, Svensson O, Gardulf A. Early individualized postoperative occupational therapy training in 100 patients improves ADL after hip fracture: A randomized trial. Acta Orthopaedica Scandinavica. 2004;75(2):177-83.

47. Hagsten B, Svensson O, Gardulf A. Health-related quality of life and self-reported ability concerning ADL and IADL after hip fracture: A randomized trial. Acta Orthopaedica. 2006;77(1):114-9.

48. Fjaertoft H, Indredavik B, Johnsen R, Lydersen S. Acute stroke unit care combined with early supported discharge. Long-term effects on quality of life. A randomized controlled trial. Clinical Rehabilitation. 2004;18(5):580-6. PubMed PMID: 15293492.

49. Fjærtoft H, Rohweder G, Indredavik B. Stroke unit care combined with early supported discharge improves 5-year outcome: a randomized controlled trial. Stroke (00392499). 2011;42(6):1707-11. PubMed PMID: 2011069121. Language: English. Entry Date: 20110826. Revision Date: 20110826. Publication Type: journal article.

50. Hendriks MRC, Bleijlevens MHC, van Haastregt JCM, Crebolder HFJM, Diederiks JPM, Evers SMAA, et al. Lack of effectiveness of a multidisciplinary fall-prevention program in elderly people at risk: a randomized, controlled trial. Journal of the American Geriatrics Society. 2008;56(8):1390-7. PubMed PMID: 18662214.

51. Zidén L, Frändin K, Kreuter M. Home rehabilitation after hip fracture. A randomized controlled study on balance confidence, physical function and everyday activities. Clinical Rehabilitation. 2008;22(12):1019-33. PubMed PMID: 2010142193. Language: English. Entry Date: 20090403. Revision Date: 20091218. Publication Type: journal article.

52. Eyres L, Unsworth CA. Occupational therapy in acute hospitals: The effectiveness of a pilot program to maintain occupational performance in older clients. Australian Occupational Therapy Journal. 2005;52(3):218-24.

53. Hinkka K, Karppi S-L, Pohjolainen T, Rantanen T, Puukka P, Tilvis R. Network-based geriatric rehabilitation for frail elderly people: feasibility and effects on subjective health and pain at one year. Journal of Rehabilitation Medicine. 2007;39(6):473-8. PubMed PMID: 17624482.

54. Lannin NA, Clemson L, McCluskey A, Lin CWC, Cameron ID, Barras S. Feasibility and results of a randomised pilot-study of pre-discharge occupational therapy home visits. BMC Health Services Research. 2007;7.

55. Clark F. Embedding health-promoting changes into the daily lives of independent-living older adults: long term follow-up of occupational therapy intervention. Journals of Gerontology. 2001;56(1):60-3.

56. Clark F, Azen SP, Zemke R, Jackson J, Carlson M, Mandel D, et al. Occupational therapy for independent-living older adults: a randomized controlled trial. JAMA: Journal of the American Medical Association. 1997;278(16):1321-6. PubMed PMID: 1998029119. Language: English. Entry Date: 19980501. Revision Date: 20091218. Publication Type: journal article.

57. de Craen A, Gussekloo J, Blauw G, Willems C, Westendorp R. Randomised controlled trial of unsolicited occupational therapy in community-dwelling elderly people: the LOTIS trial. PLoS Clinical Trials. 2006;1(1):0001-6.

58. Liddle J, March L, Carfrae B, Finnegan T, Druce J, Schwarz J, et al. Can occupational therapy intervention play a part in maintaining independence and quality of life in older people? A randomised controlled trial. Australian & New Zealand Journal of Public Health. 1996;20(6):574-8. PubMed PMID: 9117961.

59. Pitkala K. The effectiveness of day hospital care on home care patients. Journal of the American Geriatrics Society. 1998;46(9):1086-90. PubMed PMID: 9736100.

60. Nikolaus T, Specht-Leible N, Bach M, Oster P, Schlierf G. A randomized trial of comprehensive geriatric assessment and home intervention in the care of hospitalized patients. Age & Ageing. 1999;28(6):543-50. PubMed PMID: 10604506.

61. Ollonqvist K, Aaltonen T, Karppi S-L, Hinkka K, Pontinen S. Network-based rehabilitation increases formal support of frail elderly home-dwelling persons in Finland: randomised controlled trial. Health & Social Care in the Community. 2008;16(2):115-25. PubMed PMID: 18290977.

62. Clemson L, Singh MF, Bundy A, Cumming RG, Weissel E, Munro J, et al. LiFE Pilot Study: A randomised trial of balance and strength training embedded in daily life activity to reduce falls in older adults. Australian Occupational Therapy Journal. 2010;57(1):42-50. PubMed PMID: 20854564.

63. Vass M, Avlund K, Lauridsen J, Hendriksen C. Feasible model for prevention of functional decline in older people: municipality-randomized, controlled trial. Journal of the American Geriatrics Society. 2005;53(4):563-8. PubMed PMID: 2005112382. Language: English. Entry Date: 20050729. Revision Date: 20101231. Publication Type: journal article.

64. Avlund K, Jepsen E, Vass M, Lundemark H. Effects of comprehensive follow-up home visits after hospitalization on functional ability and readmissions among old patients. A randomized controlled study. Scandinavian Journal of Occupational Therapy. 2002;9(1):17-22.

65. Crotty M, Whitehead C, Miller M, Gray S. Patient and caregiver outcomes 12 months after home-based therapy for hip fracture: a randomized controlled trial. Archives of Physical Medicine & Rehabilitation. 2003;84(8):1237-9. PubMed PMID: 12917867.

66. Crotty M, Whitehead CH, Gray S, Finucane PM. Early discharge and home rehabilitation after hip fracture achieves functional improvements: a randomized controlled trial. Clinical Rehabilitation. 2002;16(4):406-13. PubMed PMID: 12061475.

67. Tucker MA, Davison JG, Ogle SJ. Day hospital rehabilitation--effectiveness and cost in the elderly: a randomised controlled trial. British Medical Journal Clinical Research Ed. 1984;289(6453):1209-12. PubMed PMID: 6437487; PubMed Central PMCID: PMCPMC1443331.

68. Bautz-Holter E, Sveen U, Rygh J, Rodgers H, Wyller TB. Early supported discharge of patients with acute stroke: a randomized controlled trial. Disability & Rehabilitation. 2002;24(7):348-55. PubMed PMID: 12022784.

69. Clarke CE, Furmston A, Morgan E, Patel S, Sackley C, Walker M, et al. Pilot randomised controlled trial of occupational therapy to optimise independence in Parkinson's disease: the PD OT trial. Journal of Neurology, Neurosurgery & Psychiatry. 2009;80(9):976-8. PubMed PMID: 18339727.

70. Logan PA, Coupland CAC, Gladman JRF, Sahota O, Stoner-Hobbs V, Robertson K, et al. Community falls prevention for people who call an emergency ambulance after a fall: randomised controlled trial. BMJ. 2010;340:c2102. PubMed PMID: 20460331; PubMed Central PMCID: PMCPMC2868162.

71. Rodgers H, Soutter J, Kaiser W, Pearson P, Dobson R, Skilbeck C, et al. Early supported hospital discharge following acute stroke: Pilot study results. Clinical Rehabilitation. 1997;11(4):280-7.

72. Abizanda P, Leon M, Dominguez-Martin L, Lozano-Berrio V, Romero L, Luengo C, et al. Effects of a short-term occupational therapy intervention in an acute geriatric unit. A randomized clinical trial. Maturitas. 2011;69(3):273-8. PubMed PMID: 21600709.

73. Askim T, Rohweder G, Lydersen S, Indredavik B. Evaluation of an extended stroke unit service with early supported discharge for patients living in a rural community. A randomized controlled trial. Clinical Rehabilitation. 2004;18(3):238-48. PubMed PMID: 15137554.

74. Close J, Ellis M, Hooper R, Glucksman E, Jackson S, Swift C. Prevention of falls in the elderly trial (PROFET): a randomised controlled trial. Lancet. 1999;353(9147):93-7. PubMed PMID: 10023893.

75. Fagerberg B, Claesson L, Gosman-Hedstrom G, Blomstrand C. Effect of acute stroke unit care integrated with care continuum versus conventional treatment: A randomized 1-year study of elderly patients: the Goteborg 70+ Stroke Study. Stroke. 2000;31(11):2578-84. PubMed PMID: 11062278.

76. Fjaertoft H, Indredavik B, Lydersen S. Stroke unit care combined with early supported discharge: long-term follow-up of a randomized controlled trial. Stroke. 2003;34(11):2687-91. PubMed PMID: 14576376.

77. Hendriksen H, Harrison RA. Occupational therapy in accident and emergency departments: a randomized controlled trial. Journal of Advanced Nursing. 2001;36(6):727-32. PubMed PMID: 11903702.

78. Indredavik B, Fjaertoft H, Ekeberg G, Loge AD, Morch B. Benefit of an extended stroke unit service with early supported discharge: A randomized, controlled trial. Stroke. 2000;31(12):2989-94. PubMed PMID: 11108761.

79. Ronning OM, Guldvog B. Outcome of subacute stroke rehabilitation: a randomized controlled trial. Stroke (00392499). 1998;29(4):779-84. PubMed PMID: 1998064729. Language: English. Entry Date: 19981001. Revision Date: 20091218. Publication Type: journal article.

80. Shepperd S, Harwood D, Jenkinson C, Gray A, Vessey G, Morgan P. Randomised controlled trial comparing hospital at home care with inpatient hospital care. I: three month follow up of health outcomes. BMJ: British Medical Journal (International Edition). 1998;316(7147):1786-91. doi: 10.1136/bmj.316.7147.1786. PubMed PMID: 1998045983. Language: English. Entry Date: 19980801. Revision Date: 20120120. Publication Type: journal article.

81. Pardessus V, Puisieux F, Di PC, Gaudefroy C, Thevenon A, Dewailly P. Benefits of home visits for falls and autonomy in the elderly: a randomized trial study. American Journal of Physical Medicine and Rehabilitation. 2002;81(4):247-52. PubMed PMID: 0036228.

82. Demers L, Desrosiers J, Nikolova R, Robichaud L, Bravo G. Responsiveness of Mobility, Daily Living, and Instrumental Activities of Daily Living Outcome Measures for Geriatric Rehabilitation. Archives of Physical Medicine and Rehabilitation. 2010;91(2):233-40.

83. Hebert R, Carrier R, Bilodeau A. THE FUNCTIONAL AUTONOMY MEASUREMENT SYSTEM (SMAF): DESCRIPTION AND VALIDATION OF AN INSTRUMENT FOR THE MEASUREMENT OF HANDICAPS. Age and Ageing. 1988;17(5):293-302. doi: 10.1093/ageing/17.5.293.

84. Hebert R, Spiegelhalter DJ, Brayne C. Setting the minimal metrically detectable change on disability rating scales. Archives of Physical Medicine and Rehabilitation. 1997;78(12):1305-8.

85. Desrosiers J, Rochette A, Noreau L, Bravo G, Hebert R, Boutin C. Comparison of two functional independence scales with a participation measure in post-stroke rehabilitation. Archives of Gerontology and Geriatrics. 2003;37(2):157-72.

86. Mercier L, Audet T, Hebert R, Rochette A, Dubois MF. Impact of motor, cognitive, and perceptual disorders on ability to perform activities of daily living after stroke. Stroke. 2001;32(11):2602-8.

87. Albert SM, Bear-Lehman J, Burkhardt A, Merete-Roa B, Noboa-Lemonier R, Teresi J. Variation in sources of clinician-rated and self-rated instrumental activities of daily living disability. Journals of Gerontology - Series A Biological Sciences and Medical Sciences. 2006;61(8):826-31.

88. Doble SE, Fisk JD, MacPherson KM, Fisher AG, Rockwood K. Measuring functional compentence in older persons with Alzheimer's Disease. International Psychogeriatrics. 1997;9(1):25-38.

89. Doble SE, Fisk JD, Rockwood K. Assessing the ADL functioning of persons with Alzheimer's disease: comparison of family informants' ratings and performance-based assessment findings. International psychogeriatrics / IPA. 1999;11(4):399-409.

90. Fioravanti AM, Bordignon CM, Pettit SM, Woodhouse LJ, Ansley BJ. Comparing the responsiveness of the assessment of motor and process skills and the functional independence measure. Canadian journal of occupational therapy Revue canadienne d'ergothérapie. 2012;79(3):167-74.

91. Doble SE, Fisk JD, Lewis N, Rockwood K. Test-retest reliability of the assessment of motor and process skills in elderly adults. Occupational Therapy Journal of Research. 1999;19(3):203-15.

92. Carter J, Mant F, Mant J, Wade D, Winner S. Comparison of postal version of the Frenchay Activities Index with interviewer-administered version for use in people with stroke. Clinical Rehabilitation. 1997;11(2):131-8. PubMed PMID: 1997045725. Language: English. Entry Date: 19971201. Revision Date: 20091218. Publication Type: journal article.

93. Wilkinson PR, Wolfe CD, Warburton FG, Rudd AG, Howard RS, Ross-Russell RW, et al. Longer term quality of life and outcome in stroke patients: is the Barthel index alone an adequate measure of outcome? Quality in health care : QHC. 1997;6(3):125-30.

94. Hall KM, Hamilton BB, Gordon WA, Zasler ND. Characteristics and comparisons of functional assessment indices: Disability Rating Scale, Functional Independence Measure, and Functional Assessment Measure. Journal of Head Trauma Rehabilitation. 1993;8(2):60-74. PubMed PMID: 1993165916. Language: English. Entry Date: 19930801. Revision Date: 20091218. Publication Type: journal article.

95. Fricke J, Unsworth CA. Inter-rater reliability of the original and modified Barthel Index, and a comparison with the Functional Independence Measure. Australian Occupational Therapy Journal. 1996;44(1):22-9. PubMed PMID: 1998001535. Language: English. Entry Date: 19980101. Revision Date: 20091218. Publication Type: journal article.

96. Hobart J, Lamping D, Freeman J, Langdon D, McLellan D, Greenwood R, et al. Evidence-based measurement: Which disability scale for neurologic rehabilitation? Neurology. 2001;57:639-44.

97. Jette DU, Warren RL, Wirtalla C. Functional independence domains in patients receiving rehabilitation in skilled nursing facilities: evaluation of psychometric properties. Archives of Physical Medicine & Rehabilitation. 2005;86(6):1089. PubMed PMID: 2009003666. Language: English. Entry Date: 20051104. Revision Date: 20091218. Publication Type: journal article.

98. Reuben DB, Valle LA, Hays RD, Siu AL. Measuring physical function in community-dwelling older persons: a comparison of self-administered, interviewer-administered, and performance-based measures. Journal of the American Geriatrics Society. 1995;43(1):17-23. PubMed PMID: 7806733.

99. Dennis M, O'Rourke S, Lewis S, Sharpe M, Warlow C. Emotional outcomes after stroke: Factors associated with poor outcome. Journal of Neurology Neurosurgery and Psychiatry. 2000;68(1):47-52.

100. Barer DH, Murphy JJ. Scaling the Barthel: a 10-point hierarchical version of the activities of daily living index for use with stroke patients. Clinical Rehabilitation. 1993;7(4):271-7. PubMed PMID: 1994188021. Language: English. Entry Date: 19940701. Revision Date: 20091218. Publication Type: journal article.

101. Yohannes AM, Roomi J, Waters K, Connolly MJ. A comparison of the Barthel index and Nottingham extended activities of daily living scale in the assessment of disability in chronic airflow limitation in old age. Age & Ageing. 1998;27(3):369-74. PubMed PMID: 1998058399. Language: English. Entry Date: 19981001. Revision Date: 20091218. Publication Type: journal article.

102. de Morton NA, Keating JL, Davidson M. Rasch analysis of the Barthel Index in the assessment of hospitalized older patients after admission for an acute medical condition. Archives of Physical Medicine & Rehabilitation. 2008;89(4):641-7. PubMed PMID: 2009893205. Language: English. Entry Date: 20080516. Revision Date: 20091218. Publication Type: journal article.

103. Wright J, Cross J, Lamb S. Physiotherapy outcome measures for rehabilitation of elderly people: responsiveness to change of the Rivermead Mobility Index and Barthel Index. Physiotherapy. 1998;84(5):216-21. PubMed PMID: 1998056509. Language: English. Entry Date: 19980901. Revision Date: 20091218. Publication Type: journal article.

104. Harwood RH, Ebrahim S. Measuring the outcomes of day hospital attendance: a comparison of the Barthel Index and London Handicap Scale. Clinical Rehabilitation. 2000;14(5):527-31. PubMed PMID: 2001017865. Language: English. Entry Date: 20010223. Revision Date: 20091218. Publication Type: journal article.

105. Bond M, Clark M. Clinical applications of the Adelaide Activities Profile. Clinical Rehabilitation. 1998;12(3):228-37. PubMed PMID: 1999002209. Language: English. Entry Date: 19990101. Revision Date: 20091218. Publication Type: journal article.

106. Clark M, Bond M. The Adelaide Activities Profile: A measure of the lifestyle activities of elderly people. Aging - Clinical and Experimental Research. 1995;7:174-84.

107. Newson RS, Kemps EB. General lifestyle activities as a predictor of current cognition and cognitive change in older adults: a cross-sectional and longitudinal examination. Journals of Gerontology Series B: Psychological Sciences & Social Sciences. 2005;60B(3):P113-20. PubMed PMID: 2005099693. Language: English. Entry Date: 20050624. Revision Date: 20091218. Publication Type: journal article.

108. Gladman JR, Lincoln NB, Adams SA. Use of the extended ADL scale with stroke patients. Age & Ageing. 1993;22(6):419-24. doi: 10.1093/ageing/22.6.419. PubMed PMID: 2011146094. Language: English. Entry Date: 20110610. Revision Date: 20120302. Publication Type: journal article.

109. Mahoney RI, Barthel DW. Barthel Index (BI). Surya Shah, PhD, OTD, MEd, OTR, FAOTA, Professor Occupational Therapy and Neurology, Visiting Professor Neurorehabilitation, University of Tennessee Health Sciences Center, 930 Madison, Suite 601, Memphis, TN 38163; 1965. p. 1 p.

110. Collin C, Wade DT, Davies S, Horne V. The Barthel ADL Index: A reliability study. International Disability Studies. 1988;10(2):61-3.

111. Shah S, Vanclay F, Cooper B. Improving the sensitivity of the Barthel Index for stroke rehabilitation. Journal of Clinical Epidemiology. 1989;42(8):703-9. doi: http://dx.doi.org/10.1016/0895-4356(89)90065-6.

112. Vergauwen K, Huijnen IPJ, Kos D, Van de Velde D, van Eupen I, Meeus M. Assessment of activity limitations and participation restrictions with persons with chronic fatigue syndrome: a systematic review. Disability and Rehabilitation. 0(0):1-11. doi: doi:10.3109/09638288.2014.978507. PubMed PMID: 25365699.

113. De Vet HCW, Terwee CB, Mokkink LB, Knol DL. Measurement in Medicine. Cambridge: Cambridge University Press; 2011.

114. Hebert R, Guilbeault J, Pinsonnault E. Functional Autonomy Measurement System: User Guide Sherbrooke: Sherbrooke Health Expertise Centre, 2002.

115. Duncan E, Murray J. The barriers and facilitators to routine outcome measurement by allied health professionals in practice: a systematic review. BMC Health Services Research. 2012;12(1):96. PubMed PMID: doi:10.1186/1472-6963-12-9 6.

116. Bernspang B, Fisher AG. Differences between persons with right or left cerebral vascular accident on the assessment of motor and process skills. Archives of Physical Medicine & Rehabilitation. 1995;76(12):1144-51. PubMed PMID: 1996017520. Language: English. Entry Date: 19960601. Revision Date: 20091218. Publication Type: journal article.

117. Duran LJ, Fisher AG. Male and female performance on the assessment of motor and process skills. Archives of Physical Medicine and Rehabilitation. 1996;77(10):1019-24.

118. Hartman ML, Fisher AG, Duran L. Assessment of functional ability of people with Alzheimer's disease. Scandinavian Journal of Occupational Therapy. 1999;6(3):111-8. PubMed PMID: 2000040808. Language: English. Entry Date: 20000601. Revision Date: 20091218. Publication Type: journal article.

119. Kottorp A, Bernspang B, Fisher AG. Validity of a performance assessment of activities of daily living for people with developmental disabilities. Journal of Intellectual Disability Research. 2003;47(8):597-605.

120. Kottorp A. The use of the Assessment of Motor and Process Skills (AMPS) in predicting need of assistance for adults with mental retardation. OTJR Occupation, Participation and Health. 2008;28(2):72-80.

121. McNulty MC, Fisher AG. Validity of using the Assessment of Motor and Process Skills to estimate overall home safety in persons with psychiatric conditions. The American journal of occupational therapy : official publication of the American Occupational Therapy Association. 2001;55(6):649-55.

122. Merritt BK, Fisher AG. Gender differences in the performance of activities of daily living. Archives of Physical Medicine and Rehabilitation. 2003;84(12):1872-7. doi: http://dx.doi.org/10.1016/S0003-9993(03)00483-0.

123. Merritt BK. Utilizing AMPS ability measures to predict level of community dependence. Scandinavian Journal of Occupational Therapy. 2010;17(1):70-6.

124. Merritt BK. Validity of using the Assessment of Motor and Process Skills to determine the need for assistance. American Journal of Occupational Therapy. 2011;65(6):643-50.

125. Pan A, Fisher AG. The assessment of motor and process skills of persons with psychiatric disorders. American Journal of Occupational Therapy. 1994;48(9):775-80. PubMed PMID: 1999064614. Language: English. Entry Date: 19991001. Revision Date: 20091218. Publication Type: journal article.

126. Poole JL, Atanasoff G, Pelsor JC, Sibbitt Jr WL. Comparison of a self-report and performance-based test of disability in people with systemic lupus erythematosus. Disability and Rehabilitation. 2006;28(10):653-8.

127. Robinson SE, Fisher AG. A study to examine the relationship of the assessment of motor and process skills (AMPS) to other tests of cognition and function. British Journal of Occupational Therapy. 1996;59(6):260-3. PubMed PMID: 1996031027. Language: English. Entry Date: 19961001. Revision Date: 20091218. Publication Type: journal article.

128. Robinson SE, Fisher AG. Functional and cognitive differences between cognitively-well people and people with dementia. British Journal of Occupational Therapy. 1999;62(10):466-71. PubMed PMID: 2000000954. Language: English. Entry Date: 20000101. Revision Date: 20091218. Publication Type: journal article.

129. Stauffer LM, Fisher AG, Duran L. ADL performance of black Americans and white Americans on the assessment of motor and process skills. American Journal of Occupational Therapy. 2000;54(6):607-13. PubMed PMID: 2001026671. Language: English. Entry Date: 20010323. Revision Date: 20091218. Publication Type: journal article.

130. White BP, Mulligan SE. Behavioral and physiologic response measures of occupational task performance: A preliminary comparison between typical children and children with attention disorder. American Journal of Occupational Therapy. 2005;59(4):426-36.

131. White BP, Mulligan S, Merrill K, Wright J. An examination of the relationships between motor and process skills and scores on the sensory profile. The American journal of occupational therapy : official publication of the American Occupational Therapy Association. 2007;61(2):154-60.

132. Ahmed S, Mayo NE, Higgins J, Salbach NM, Finch L, Wood-Dauphinée SL. The Stroke Rehabilitation Assessment of Movement (STREAM): a comparison with other measures used to evaluate effects of stroke and rehabilitation. Physical Therapy. 2003;83(7):617-30. PubMed PMID: 2003122785. Language: English. Entry Date: 20030912. Revision Date: 20091218. Publication Type: journal article.

133. Filiatrault J, Arsenault AB, Dutil E, Bourbonnais D. Motor function and activities of daily living assessments: a study of three tests for persons with hemiplegia. American Journal of Occupational Therapy. 1991;45(9):806-10. PubMed PMID: 1991129417. Language: English. Entry Date: 19911001. Revision Date: 20091218. Publication Type: journal article.

134. Khan F, Pallant JF, Turner-Stokes L. Use of goal attainment scaling in inpatient rehabilitation for persons with multiple sclerosis. Archives of Physical Medicine & Rehabilitation. 2008;89(4):652-9. PubMed PMID: 2009893207. Language: English. Entry Date: 20080516. Revision Date: 20091218. Publication Type: journal article.

135. Kwon S, Hartzema AG, Duncan PW, Lai S. Disability measures in stroke: relationship among the Barthel Index, the Functional Independence Measure, and the Modified Rankin Scale. Stroke (00392499). 2004;35(4):918-23. PubMed PMID: 2004160802. Language: English. Entry Date: 20041001. Revision Date: 20091218. Publication Type: journal article.

136. Wallace D, Duncan PW, Lai SM. Comparison of the responsiveness of the Barthel Index and the Motor Component of the Functional Independence Measure in stroke: The impact of using different methods for measuring responsiveness. Journal of Clinical Epidemiology. 2002;55(9):922-8.

137. Wellwood I, Dennis MS, Warlow CP. A comparison of the Barthel Index and the OPCS Disability Instrument used to measure outcome after acute stroke. Age & Ageing. 1995;24(1):54-7. PubMed PMID: 1997012126. Language: English. Entry Date: 19970401. Revision Date: 20091218. Publication Type: journal article.

138. Al-Khawaja I, Wade DT, Turner F. The Barthel Index and its relationship to nursing dependency in rehabilitation. Clinical Rehabilitation. 1997;11(4):335-7. PubMed PMID: 1998015212. Language: English. Entry Date: 19980301. Revision Date: 20091218. Publication Type: journal article.

139. Brazil L, Thomas R, Laing R, Hines F, Guerrero D, Ashley S, et al. Verbally administered Barthel Index as functional assessment in brain tumour patients. Journal of Neuro-Oncology. 1997;34(2):187-92.

140. Green J, Forster A, Young J. A test-retest reliability study of the Barthel Index, the Rivermead Mobility Index, the Nottingham Extended Activities of Daily Living Scale and the Frenchay Activities Index in stroke patients. Disability and Rehabilitation. 2001;23(15):670-6.

141. Gompertz P, Pound P, Ebrahim S. Validity of the extended activities of daily living scale. Clinical Rehabilitation. 1994;8(4):275-80. PubMed PMID: 1995002700. Language: English. Entry Date: 19950201. Revision Date: 20091218. Publication Type: journal article.

142. Hartigan I, O'Mahony D. The Barthel Index: comparing inter-rater reliability between Nurses and Doctors in an older adult rehabilitation unit. Applied Nursing Research. 2011;24(1):e1-7. doi: 10.1016/j.apnr.2009.11.002. PubMed PMID: 2010932528. Language: English. Entry Date: 20110325. Revision Date: 20110805. Publication Type: journal article.

143. Hatfield A, Hunt S, Wade DT. The Northwick Park Dependency Score and its relationship to nursing hours in neurological rehabilitation. Journal of Rehabilitation Medicine (Taylor & Francis Ltd). 2003;35(3):116-20. PubMed PMID: 2003134703. Language: English. Entry Date: 20031017. Revision Date: 20100604. Publication Type: journal article.

144. Hobart J, Cano S, Thompson A. Effect sizes can be misleading: Is it time to change the way we measure change? Journal of Neurology, Neurosurgery and Psychiatry. 2010;81(9):1044-8.

145. Houlden H, Edwards M, McNeil J, Greenwood R. Use of the Barthel Index and the Functional Independence Measure during early inpatient rehabilitation after single incident brain injury. Clinical Rehabilitation. 2006;20(2):153-9. PubMed PMID: 2009140867. Language: English. Entry Date: 20060630. Revision Date: 20091218. Publication Type: journal article.

146. Kidd D, Stewart G, Baldry J, Johnson J, Rossiter D, Petruckevitch A, et al. The Functional Independence Measure: A comparative validity and reliability study. Disability and Rehabilitation. 1995;17(1):10-4.

147. Parker S, Du X, Bardsley MJ, Goodfellow J, Cooper RG, Cleary R, et al. Measuring outcomes in care of the elderly. Journal of the Royal College of Physicians of London. 1994;28(5):428-33.

148. Richards SH, Peters TJ, Coast J, Gunnell DJ, Darlow M, Pounsford J. Inter-rater reliability of the Barthel ADL Index: how does a researcher compare to a nurse? Clinical Rehabilitation. 2000;14(1):72-8. PubMed PMID: 2000031262. Language: English. Entry Date: 20000501. Revision Date: 20091218. Publication Type: journal article.

149. Sarker SJ, Rudd AG, Douiri A, Wolfe CD. Comparison of 2 Extended Activities of Daily Living Scales With the Barthel Index and Predictors of Their Outcomes: Cohort Study Within the South London Stroke Register (SLSR). Stroke (00392499). 2012;43(5):1362-9. PubMed PMID: 2011535115. Language: English. Entry Date: In Process. Revision Date: 20120511. Publication Type: journal article. Journal Subset: Allied Health.

150. Van Der Putten JJMF, Hobart JC, Freeman JA, Thompson AJ. Measuring change in disability after inpatient rehabilitation: Comparison of the responsiveness of the Barthel Index and the Functional Independence Measure. Journal of Neurology Neurosurgery and Psychiatry. 1999;66(4):480-4.

151. Wade DT, Collin C. The Barthel ADL Index: A standard measure of physical disability? International Disability Studies. 1988;10(2):64-7.

152. Wade DT, Hewer RL. Functional abilities after stroke: Measurement, natural history and prognosis. Journal of Neurology Neurosurgery and Psychiatry. 1987;50(2):177-82.

153. Bodiam C. The use of the Canadian Occupational Performance Measure for the assessment of outcome on a neurorehabilitation unit. British Journal of Occupational Therapy. 1999;62(3):123-6. PubMed PMID: 1999041456. Language: English. Entry Date: 19990601. Revision Date: 20091218. Publication Type: journal article.

154. Carpenter L, Baker GA, Tyldesley B. The use of the Canadian Occupational Performance Measure as an outcome of a pain management program. Canadian Journal of Occupational Therapy. 2001;68(1):16-22. PubMed PMID: 2001051917. Language: English. Entry Date: 20010608. Revision Date: 20091218. Publication Type: journal article.

155. Case-Smith J. Outcomes in hand rehabilitation using occupation therapy services. American Journal of Occupational Therapy. 2003;57(5):499-506. PubMed PMID: 2004051074. Language: English. Entry Date: 20040402. Revision Date: 20091218. Publication Type: journal article.

156. Chan CCH, Lee TMC. Validity of the Canadian Occupational Performance Measure. Occupational Therapy International. 1997;4(3):229-47. PubMed PMID: 1998049510. Language: English. Entry Date: 19980801. Revision Date: 20091218. Publication Type: journal article.

157. Donnelly C, Eng JJ, Hall J, Alford L, Giachino R, Norton K, et al. Client-centred assessment and the identification of meaningful treatment goals for individuals with a spinal cord injury. Spinal Cord. 2004;42(5):302-7. PubMed PMID: 2009033982. Language: English. Entry Date: 20051014. Revision Date: 20091218. Publication Type: journal article.

158. Edwards M, Baptiste S, Stratford PW, Law M. Recovery after hip fracture: what can we learn from the Canadian Occupational Performance Measure? American Journal of Occupational Therapy. 2007;61(3):335-44. PubMed PMID: 2009607610. Language: English. Entry Date: 20070824. Revision Date: 20091218. Publication Type: journal article.

159. Jenkinson N, Ownsworth T, Shum D. Utility of the Canadian Occupational Performance Measure in community-based brain injury rehabilitation. Brain Injury. 2007;21(12):1283-94. PubMed PMID: 2009767014. Language: English. Entry Date: 20080516. Revision Date: 20091218. Publication Type: journal article.

160. McColl MA, Paterson M, Davies D, Doubt L, Law M. Validity and community utility of the Canadian Occupational Performance Measure. Canadian Journal of Occupational Therapy. 2000;67(1):22-30. PubMed PMID: 2000023137. Language: English. Entry Date: 20000401. Revision Date: 20091218. Publication Type: journal article.

161. Law M, Polatajko H, Pollock N, McColl MA, Carswell A, Baptiste S. Pilot testing of the Canadian Occupational Performance Measure: clinical and measurement issues. Canadian Journal of Occupational Therapy. 1994;61(4):191-7. PubMed PMID: 1994196939. Language: English. Entry Date: 19941101. Revision Date: 20091218. Publication Type: journal article.

162. Ripat J, Etcheverry E, Cooper J, Tate R. A comparison of the Canadian Occupational Performance Measure and the Health Assessment Questionnaire. Canadian Journal of Occupational Therapy. 2001;68(4):247-53. PubMed PMID: 2002022084. Language: English. Entry Date: 20020201. Revision Date: 20091218. Publication Type: journal article.

163. Rochman DL, Ray SA, Kulich RJ, Mehta NR, Driscoll S. Validity and utility of the Canadian occupational performance measure as an outcome measure in a craniofacial pain center. OTJR Occupation, Participation and Health. 2008;28(1):4-11.

164. Sewell L, Singh SJ. The Canadian Occupational Performance Measure: is it a reliable measure in clients with chronic obstructive pulmonary disease? British Journal of Occupational Therapy. 2001;64(6):305-10. PubMed PMID: 2001097877. Language: English. Entry Date: 20011026. Revision Date: 20091218. Publication Type: journal article.

165. Stuber CJ, Nelson DL. Convergent validity of three occupational self-assessments. Physical & Occupational Therapy in Geriatrics. 2010;28(1):13-21. doi: 10.3109/02703180903189260. PubMed PMID: 2010613166. Language: English. Entry Date: 20100514. Revision Date: 20100514. Publication Type: journal article.

166. van Huet H, Williams D. Self-beliefs about pain and occupational performance: a comparison of two measures used in a pain management program. OTJR: Occupation, Participation & Health. 2007;27(1):4-12. PubMed PMID: 2009521532. Language: English. Entry Date: 20080411. Revision Date: 20091218. Publication Type: journal article.

167. Walsh DA, Kelly SJ, Johnson PS, Rajkumar S, Bennetts K. Performance problems of patients with chronic low-back pain and the measurement of patient-centered outcome. Spine. 2004;29(1):87-93. PubMed PMID: 2004132497. Language: English. Entry Date: 20040806. Revision Date: 20100122. Publication Type: journal article.

168. Holbrook M, Skilbeck CE. AN ACTIVITIES INDEX FOR USE WITH STROKE PATIENTS. Age and Ageing. 1983;12(2):166-70. doi: 10.1093/ageing/12.2.166.

169. Patel MD, Tilling K, Lawrence E, Rudd AG, Wolfe CDA, McKevitt C. Relationships between long-term stroke disability, handicap and health-related quality of life. Age and Ageing. 2006;35(3):273-9.

170. Turnbull JC, Kersten P, Habib M, McLellan L, Mullee MA, George S. Validation of the Frenchay Activities Index in a general population aged 16 years and older. Archives of Physical Medicine & Rehabilitation. 2000;81(8):1034-8. PubMed PMID: 2000060396. Language: English. Entry Date: 20001001. Revision Date: 20091218. Publication Type: journal article.

171. Corrigan JD, Smith-Knapp K, Granger CV. Validity of the Functional Independence Measure for persons with traumatic brain injury. Archives of Physical Medicine & Rehabilitation. 1997;78(8):828-34. PubMed PMID: 1998007604. Language: English. Entry Date: 19980201. Revision Date: 20091218. Publication Type: journal article.

172. Dodds TA, Martin DP, Stolov WC, Deyo RA. A validation of the Functional Independence Measurement and its performance among rehabilitation inpatients. Archives of Physical Medicine & Rehabilitation. 1993;74(5):531-6. PubMed PMID: 1993163631. Language: English. Entry Date: 19930701. Revision Date: 20091218. Publication Type: journal article.

173. Glenny C, Stolee P, Husted J, Thompson M, Berg K. Comparison of the responsiveness of the FIM and the interRAI Post Acute Care assessment instrument in rehabilitation of older adults. Archives of Physical Medicine & Rehabilitation. 2010;91(7):1038-43. doi: 10.1016/j.apmr.2010.03.014. PubMed PMID: 2010707697. Language: English. Entry Date: 20100903. Revision Date: 20100903. Publication Type: journal article.

174. Graves DE. The construct validity and explanatory power of the AISA Motor Score and the FIM: implications for theoretical models of spinal cord injury. Topics in Spinal Cord Injury Rehabilitation. 2005;10(4):65-74. PubMed PMID: 2009001602. Language: English. Entry Date: 20051104. Revision Date: 20120427. Publication Type: journal article.

175. Hamilton BB, Granger CV. Disability outcomes following inpatient rehabilitation for stroke. Physical Therapy. 1994;74(5):494-503. PubMed PMID: 1994186628. Language: English. Entry Date: 19940601. Revision Date: 20091218. Publication Type: journal article.

176. Heinemann AW, Linacre JM, Wright BD, Hamilton BB, Granger C. Relationships between impairment and physical disability as measured by the Functional Independence Measure. Archives of Physical Medicine & Rehabilitation. 1993;74(6):566-73. PubMed PMID: 1993165204. Language: English. Entry Date: 19930801. Revision Date: 20091218. Publication Type: journal article.

177. Heinemann AW, Linacre JM, Wright BD, Hamilton BB, Granger C. Measurement characteristics of the Functional Independence Measure. Topics in Stroke Rehabilitation. 1994;1(3):1-15. PubMed PMID: 1997023009. Language: English. Entry Date: 19970601. Revision Date: 20091218. Publication Type: journal article.

178. Heinemann AW, Kirk P, Hastie BA, Semik P, Hamilton BB, Linacre JM, et al. Relationships between disability measures and nursing effort during medical rehabilitation for patients with traumatic brain and spinal cord injury. Archives of Physical Medicine & Rehabilitation. 1997;78(2):143-9. PubMed PMID: 1997019868. Language: English. Entry Date: 19970601. Revision Date: 20091218. Publication Type: journal article.

179. Kohler F, Redmond H, Dickson H, Connolly C, Estell J. Interrater reliability of functional status scores for patients transferred from one rehabilitation setting to another. Archives of Physical Medicine & Rehabilitation. 2010;91(7):1031-7. doi: 10.1016/j.apmr.2010.03.020. PubMed PMID: 2010707696. Language: English. Entry Date: 20100903. Revision Date: 20100903. Publication Type: journal article.

180. Linacre JM, Heinemann AW, Wright BD, Granger CV, Hamilton BB. The structure and stability of the Functional Independence Measure. Archives of Physical Medicine & Rehabilitation. 1994;75(2):127-32. PubMed PMID: 1994180810. Language: English. Entry Date: 19940401. Revision Date: 20091218. Publication Type: journal article.

181. Ottenbacher KJ, Mann WC, Granger CV, Tomita M, Hurren D, Charvat B. Inter-rater agreement and stability of functional assessment in the community-based elderly. Archives of Physical Medicine & Rehabilitation. 1994;75(12):1297-301. PubMed PMID: 1995005461. Language: English. Entry Date: 19950301. Revision Date: 20091218. Publication Type: journal article.

182. Pollak N, Rheault W, Stoecker JL. Reliability and validity of the FIM for persons aged 80 years and above from a multilevel continuing care retirement community. Archives of Physical Medicine & Rehabilitation. 1996;77(10):1056-61. PubMed PMID: 1997008733. Language: English. Entry Date: 19970301. Revision Date: 20091218. Publication Type: journal article.

183. Segal ME, Ditunno JF, Staas WE. Interinstitutional agreement of individual functional independence measure (FIM) items measured at two sites on one sample of SCI patients. Paraplegia. 1993;31(10):622-31.

184. Sharrack B, Hughes RAC, Soudain S, Dunn G. The psychometric properties of clinical rating scales used in multiple sclerosis. Brain: A Journal of Neurology. 1999;122(Part 1):141-59. PubMed PMID: 1999024702. Language: English. Entry Date: 19990401. Revision Date: 20091218. Publication Type: journal article.

185. Stineman MG, Shea JA, Jette A, Tassoni CJ, Ottenbacher KJ, Fiedler R, et al. The Functional Independence Measure: tests of scaling assumptions, structure, and reliability across 20 diverse impairment categories. Archives of Physical Medicine & Rehabilitation. 1996;77(11):1101-8. PubMed PMID: 1997012371. Language: English. Entry Date: 19970401. Revision Date: 20091218. Publication Type: journal article.

186. Jette AM, Davies AR, Cleary PD, Calkins DR, Rubenstein LV, Fink A, et al. The Functional Status Questionnaire: reliability and validity when used in primary care. Journal of general internal medicine : official journal of the Society for Research and Education in Primary Care Internal Medicine. 1986;1(3):143-9.

187. Katz JN, Larson MG, Phillips CB, Fossel AH, Liang MH. Comparative measurement sensitivity of short and longer health status instruments. Medical Care. 1992;30(10):917-25.

188. Reuben DB, Rubenstein LV, Hirsch SH, Hays RD. Value of functional status as a predictor of mortality: results of a prospective study.[Erratum appears in Am J Med 1993 May;94(5):560], [Erratum appears in Am J Med 1993 Feb;94(2):232]. American Journal of Medicine. 1992;93(6):663-9. PubMed PMID: 1466363.

189. Rubenstein LM, Voelker MD, Chrischilles EA, Glenn DC, Wallace RB, Rodnitzky RL. The usefulness of the Functional Status Questionnaire and Medical Outcomes Study Short Form in Parkinson's disease research. Qual Life Res. 1998;7(4):279-90. PubMed PMID: 9610212.

190. Yarnold PR, Stille FC, Martin GJ. Cross-sectional psychometric assessment of the functional status questionnaire: Use with geriatric versus nongeriatric ambulatory medical patients. International Journal of Psychiatry in Medicine. 1995;25(4):305-17.

191. Benton N, Stewart N, Crabbe J, Robinson E, Yeoman S, McQueen FM. MRI of the wrist in early rheumatoid arthritis can be used to predict functional outcome at 6 years. Annals of the Rheumatic Diseases. 2004;63(5):555-61. PubMed PMID: 15082487; PubMed Central PMCID: PMCPMC1755000.

192. Bombardier C, Raboud J. A comparison of health-related quality-of-life measures for rheumatoid arthritis research. The Auranofin Cooperating Group. Controlled Clinical Trials. 1991;12(4 Suppl):243S-56S. PubMed PMID: 1663860.

193. Bruce B, Fries J. Longitudinal comparison of the Health Assessment Questionnaire (HAQ) and the Western Ontario and McMaster Universities Osteoarthritis Index (WOMAC). Arthritis & Rheumatism. 2004;51(5):730-7. PubMed PMID: 15478152.

194. Buchbinder R, Bombardier C, Yeung M, Tugwell P. Which outcome measures should be used in rheumatoid arthritis clinical trials? Clinical and quality-of-life measures' responsiveness to treatment in a randomized controlled trial. Arthritis & Rheumatism. 1995;38(11):1568-80. PubMed PMID: 7488277.

195. Clements PJ, Wong WK, Hurwitz EL, Furst DE, Mayes M, White B, et al. The Disability Index of the Health Assessment Questionnaire is a predictor and correlate of outcome in the high-dose versus low-dose penicillamine in systemic sclerosis trial. Arthritis & Rheumatism. 2001;44(3):653-61. PubMed PMID: 11263780.

196. Cole JC, Motivala SJ, Khanna D, Lee JY, Paulus HE, Irwin MR. Validation of single-factor structure and scoring protocol for the Health Assessment Questionnaire-Disability Index. Arthritis & Rheumatism. 2005;53(4):536-42. PubMed PMID: 16082630.

197. Cole JC, Khanna D, Clements PJ, Seibold JR, Tashkin DP, Paulus HE, et al. Single-factor scoring validation for the Health Assessment Questionnaire-Disability Index (HAQ-DI) in patients with systemic sclerosis and comparison with early rheumatoid arthritis patients. Qual Life Res. 2006;15(8):1383-94. PubMed PMID: 16826439.

198. Fries JF, Ramey DR. 'Arthritis specific' global health analog scales assess 'generic' health related quality-of-life in patients with rheumatoid arthritis. Journal of Rheumatology. 1997;24(9):1697-702.

199. Lawrence E, Pope J, Al Zahraly Z, Lalani S, Baron M. The relationship between changes in self-reported disability (measured by the Health Assessment Questionnaire - HAQ) in scleroderma and improvement of disease status in clinical practice. Clinical & Experimental Rheumatology. 2009;27(3 Suppl 54):32-7. PubMed PMID: 19796559.

200. Leigh JP, Fries JF. Predictors of disability in a longitudinal sample of patients with rheumatoid arthritis. Annals of the Rheumatic Diseases. 1992;51(5):581-7. PubMed PMID: 1535493; PubMed Central PMCID: PMCPMC1005686.

201. Marra CA, Rashidi AA, Guh D, Kopec JA, Abrahamowicz M, Esdaile JM, et al. Are indirect utility measures reliable and responsive in rheumatoid arthritis patients? Qual Life Res. 2005;14(5):1333-44. PubMed PMID: 16047508.

202. Marra CA, Woolcott JC, Kopec JA, Shojania K, Offer R, Brazier JE, et al. A comparison of generic, indirect utility measures (the HUI2, HUI3, SF-6D, and the EQ-5D) and disease-specific instruments (the RAQoL and the HAQ) in rheumatoid arthritis. Social Science & Medicine. 2005;60(7):1571-82. PubMed PMID: 15652688.

203. Milligan SE, Hom DL, Ballou SP, Persse LJ, Svilar GM, Coulton CJ. An assessment of the Health Assessment Questionnaire functional ability index among women with systemic lupus erythematosus. Journal of Rheumatology. 1993;20(6):972-6. PubMed PMID: 8350333.

204. Poole JL, Williams CA, Bloch DA, Hollak B, Spitz P. Concurrent validity of the Health Assessment Questionnaire Disability Index in Scleroderma. Arthritis care & research. 1995;8(3):189-93. PubMed PMID: 7654804.

205. Poole JL, Cordova KJ, Brower LM. Reliability and validity of a self-report of hand function in persons with rheumatoid arthritis. Journal of Hand Therapy. 2006;19(1):12-6, quiz 7. PubMed PMID: 16473729.

206. Rohekar G, Pope J. Test-retest reliability of patient global assessment and physician global assessment in rheumatoid arthritis. Journal of Rheumatology. 2009;36(10):2178-82.

207. Sultan N, Pope JE, Clements PJ, Furst DE, Siebold JR, Wong WK, et al. The health assessment questionnaire (HAQ) is strongly predictive of good outcome in early diffuse scleroderma: Results from an analysis of two randomized controlled trials in early diffuse scleroderma. Rheumatology. 2004;43(4):472-8.

208. Katz S, Ford A, Moskowitz R, Jackson B, Jaffe M. Studies of illness in the aged: The index of adl: a standardized measure of biological and psychosocial function. JAMA. 1963;185(12):914-9. doi: 10.1001/jama.1963.03060120024016.

209. Katz S, Downs T, Cash H, Grotz R. Progress in Development of the Index of ADL. The Gerontologist. 1970;10(1 Part 1):20-30. doi: 10.1093/geront/10.1_Part_1.20.

210. Klein RM, Bell B. Self-care skills: behavioral measurement with Klein-Bell ADL scale. Archives of Physical Medicine & Rehabilitation. 1982;63(7):335-8.

211. Lawton MP, Brody EM. Assessment of Older People: Self-Maintaining and Instrumental Activities of Daily Living. The Gerontologist. 1969;9(3 Part 1):179-86. doi: 10.1093/geront/9.3_Part_1.179.

212. Baker PS, Bodner EV, Allman RM. Measuring life-space mobility in community-dwelling older adults. Journal of the American Geriatrics Society. 2003;51(11):1610-4. doi: 10.1046/j.1532-5415.2003.51512.x. PubMed PMID: 2004120735. Language: English. Entry Date: 20040730. Revision Date: 20120302. Publication Type: journal article.

213. Crowe M, Andel R, Wadley VG, Okonkwo OC, Sawyer P, Allman RM. Life-space and cognitive decline in a community-based sample of African American and Caucasian older adults. Journals of Gerontology Series A: Biological Sciences & Medical Sciences. 2008;63A(11):1241-5. PubMed PMID: 2010141418. Language: English. Entry Date: 20090410. Revision Date: 20120615. Publication Type: journal article.

214. Peel C, Sawyer Baker P, Roth DL, Brown CJ, Bodner EV, Allman RM. Assessing mobility in older adults: The UAB Study of Aging Life-Space Assessment. Physical Therapy. 2005;85(10):1008-19. PubMed PMID: 2009048993. Language: English. Entry Date: 20051209. Revision Date: 20091218. Publication Type: journal article.

215. Hocking C, Williams M, Broad J, Baskett J. Sensitivity of Shah, Vanclay and Cooper's modified Barthel Index. Clinical Rehabilitation. 1999;13(2):141-7.

216. Shah S, Muncer S, Griffin J, Elliott L. The utility of the Modified Barthel Index for Traumatic Brain Injury Rehabilitation and Prognosis. Australian Occupational Therapy Journal. 2000;63(10):469-75.

217. Shah S, Muncer SJ. A comparison of rehabilitation outcome measures for traumatic brain injury. OTJR Occupation, Participation and Health. 2003;23(1):2-9.

218. Ashburn A, Hyndman D, Pickering R, Yardley L, Harris S. Predicting people with stroke at risk of falls. Age and Ageing. 2008;37(3):270-6.

219. das Nair R, Moreton BJ, Lincoln NB. Rasch analysis of the Nottingham Extended Activities of Daily Living Scale. Journal of Rehabilitation Medicine (Stiftelsen Rehabiliteringsinformation). 2011;43(10):944-50. PubMed PMID: 2011350376. Language: English. Entry Date: 20120120. Revision Date: 20120120. Publication Type: journal article.

220. Gompertz P, Pound P, Ebrahim S. The reliability of stroke outcome measures. Clinical Rehabilitation. 1993;7(4):290-6. PubMed PMID: 1994188024. Language: English. Entry Date: 19940701. Revision Date: 20091218. Publication Type: journal article.

221. Harwood RH, Ebrahim S. The validity, reliability and responsiveness of the Nottingham extended activities of daily living scale in patients undergoing total hip replacement. Disability and Rehabilitation. 2002;24(7):371-7.

222. Jacob-Lloyd HA, Dunn OM, Brain ND, Lamb SE. Effective measurement of the functional progress of stroke clients. British Journal of Occupational Therapy. 2005;68(6):253-9. PubMed PMID: 2009021326. Language: English. Entry Date: 20051202. Revision Date: 20091218. Publication Type: journal article.

223. Lincoln NB, Gladman JR. The extended activities of daily living scale: A further validation. Disability and Rehabilitation. 1992;14(1):41-3.

224. Nicholl CR, Lincoln NB, Playford ED. The reliability and validity of the Nottingham Extended Activities of Daily Living Scale in patients with multiple sclerosis. Multiple Sclerosis. 2002;8(5):372-6.

225. Nouri F, Lincoln N. An extended activities of daily living scale for stroke patients. Clinical Rehabilitation. 1987;1(4):301-5. doi: 10.1177/026921558700100409.

226. Wood-Dauphinee SL, Opzoomer MA, Williams JI, Marchand B, Spitzer WO. Assessment of global function: The reintegration to normal living index. Archives of Physical Medicine and Rehabilitation. 1988;69(8):583-90.

227. Lincoln NB, Edmans JA. A re-validation of the Rivermead ADL scale for elderly patients with stroke. Age & Ageing. 1990;19(1):19-24. PubMed PMID: 2316420.

228. Whiting S, Lincoln N. An ADL assessment for stroke patients. British Journal of Occupational Therapy. 1980;43:44-6.
